# Vessel co‐option is common in human lung metastases and mediates resistance to anti‐angiogenic therapy in preclinical lung metastasis models

**DOI:** 10.1002/path.4845

**Published:** 2016-12-29

**Authors:** Victoria L Bridgeman, Peter B Vermeulen, Shane Foo, Agnes Bilecz, Frances Daley, Eleftherios Kostaras, Mark R Nathan, Elaine Wan, Sophia Frentzas, Thomas Schweiger, Balazs Hegedus, Konrad Hoetzenecker, Ferenc Renyi‐Vamos, Elizabeth A Kuczynski, Naveen S Vasudev, James Larkin, Martin Gore, Harold F Dvorak, Sandor Paku, Robert S Kerbel, Balazs Dome, Andrew R Reynolds

**Affiliations:** ^1^ Tumour Biology Team, The Breast Cancer Now Toby Robins Research CentreThe Institute of Cancer ResearchLondonUK; ^2^ Translational Cancer Research Unit (TCRU)GZA Hospitals St AugustinusAntwerpBelgium; ^3^ 2nd Institute of PathologySemmelweis UniversityBudapestHungary; ^4^ Breast Cancer Now Histopathology Core Facility, The Royal MarsdenLondonUK; ^5^ The Royal MarsdenLondonUK; ^6^ Department of Thoracic SurgeryMedical University of ViennaViennaAustria; ^7^ Department of Thoracic Surgery, Ruhrlandklinik EssenUniversity Hospital of University Duisburg‐EssenGermany; ^8^ MTA‐SE Molecular Oncology Research GroupHungarian Academy of SciencesBudapestHungary; ^9^ Department of Thoracic SurgerySemmelweis University–National Institute of OncologyBudapestHungary; ^10^ Department of Medical BiophysicsUniversity of TorontoTorontoCanada; ^11^ Cancer Research UK Centre, Leeds Institute of Cancer and PathologySt James's University HospitalLeedsUK; ^12^ Beth Israel Deaconess Medical CenterBostonMAUSA; ^13^ 1st Department of Pathology and Experimental Cancer ResearchSemmelweis UniversityBudapestHungary; ^14^ Tumour Progression Research GroupHungarian Academy of Sciences–Semmelweis UniversityBudapestHungary; ^15^ Biological Sciences Platform, Sunnybrook Research InstituteTorontoCanada; ^16^ National Koranyi Institute of PulmonologyBudapestHungary; ^17^ Department of Biomedical Imaging and Image‐guided TherapyMedical University of ViennaAustria

**Keywords:** lung metastasis, angiogenesis, vessel co‐option, anti‐angiogenic therapy, sunitinib, drug resistance

## Abstract

Anti‐angiogenic therapies have shown limited efficacy in the clinical management of metastatic disease, including lung metastases. Moreover, the mechanisms via which tumours resist anti‐angiogenic therapies are poorly understood. Importantly, rather than utilizing angiogenesis, some metastases may instead incorporate pre‐existing vessels from surrounding tissue (vessel co‐option). As anti‐angiogenic therapies were designed to target only new blood vessel growth, vessel co‐option has been proposed as a mechanism that could drive resistance to anti‐angiogenic therapy. However, vessel co‐option has not been extensively studied in lung metastases, and its potential to mediate resistance to anti‐angiogenic therapy in lung metastases is not established. Here, we examined the mechanism of tumour vascularization in 164 human lung metastasis specimens (composed of breast, colorectal and renal cancer lung metastasis cases). We identified four distinct histopathological growth patterns (HGPs) of lung metastasis (alveolar, interstitial, perivascular cuffing, and pushing), each of which vascularized via a different mechanism. In the alveolar HGP, cancer cells invaded the alveolar air spaces, facilitating the co‐option of alveolar capillaries. In the interstitial HGP, cancer cells invaded the alveolar walls to co‐opt alveolar capillaries. In the perivascular cuffing HGP, cancer cells grew by co‐opting larger vessels of the lung. Only in the pushing HGP did the tumours vascularize by angiogenesis. Importantly, vessel co‐option occurred with high frequency, being present in >80% of the cases examined. Moreover, we provide evidence that vessel co‐option mediates resistance to the anti‐angiogenic drug sunitinib in preclinical lung metastasis models. Assuming that our interpretation of the data is correct, we conclude that vessel co‐option in lung metastases occurs through at least three distinct mechanisms, that vessel co‐option occurs frequently in lung metastases, and that vessel co‐option could mediate resistance to anti‐angiogenic therapy in lung metastases. Novel therapies designed to target both angiogenesis and vessel co‐option are therefore warranted. © 2016 The Authors. *The Journal of Pathology* published by John Wiley & Sons Ltd on behalf of Pathological Society of Great Britain and Ireland.

## Introduction

Although the progression of metastases is considered to require new blood vessel growth (angiogenesis), anti‐angiogenic drugs have shown limited efficacy in patients with metastatic disease. Metastases can be either unresponsive to anti‐angiogenic therapy from the outset (intrinsic resistance) or can develop resistance after an initial period of response (acquired resistance). The mechanisms that mediate this resistance are still poorly understood 1, 2, 3, 4, 5.

However, rather than inducing angiogenesis, it now emerges that some tumours can instead incorporate pre‐existing blood vessels from the surrounding normal tissue, a process known as vessel co‐option or vascular co‐option 5, 6, 7. For example, seminal studies on non‐small‐cell lung cancer (NSCLC) demonstrated that some NSCLCs utilize vessel co‐option instead of angiogenesis 8, 9, 10, 11, 12. In this ‘non‐angiogenic’ subtype of NSCLC, the cancer cells grow only within the alveolar air spaces. This permits intact alveolar walls to be incorporated into the tumour, allowing the tumour to co‐opt the alveolar capillaries that are contained within those alveolar walls 8, 9, 10, 11, 12. A similar presentation has been reported in some cases of human lung metastasis 13, 14, 15. In addition, we recently examined the mechanism of tumour vascularization in several preclinical models of lung metastasis. In all models examined, the lung metastases co‐opted alveolar capillaries by occupying the alveolar air spaces 16.

Given that conventional anti‐angiogenic drugs were designed only to inhibit angiogenesis, the presence of vessel co‐option in tumours may help to explain the limited efficacy of conventional anti‐angiogenic therapies 7. In support of this, vessel co‐option has now been implicated as a mechanism of resistance to anti‐angiogenic drugs in glioblastoma 17, 18, 19, hepatocellular carcinoma 20, lymph node metastases 21, liver metastases 22, and brain metastases 23, 24. However, a role for vessel co‐option in driving therapy resistance in lung metastases has not been reported.

In the current article, we describe three distinct mechanisms of vessel co‐option in human lung metastases. We also quantify the incidence of vessel co‐option across a large series of human lung metastasis cases. Finally, we utilize preclinical lung metastasis models to investigate whether vessel co‐option can mediate resistance to anti‐angiogenic therapy.

## Materials and methods

### Human samples

Formalin‐fixed paraffin‐embedded samples of human lung metastases were retrieved from archives at the St Augustinus Hospital (Antwerp, Belgium), the Medical University of Vienna (Vienna, Austria), and the National Koranyi Institute of Pulmonology (Budapest, Hungary). This initial series consisted of 193 lesions from 181 patients. Haematoxylin and eosin (H&E)‐stained sections were prepared from all cases for an initial histopathological assessment. Twenty‐nine lesions were then excluded because they were unsuitable (supplementary material, Figures S1–S3). The final series analysed consisted of 164 lesions from 158 patients (46 breast cancer metastases from 46 patients, 57 colorectal cancer metastases from 53 patients, and 61 renal cancer metastases from 59 patients). For patient details, see supplementary material, Tables S1–S3. Ethical approval was obtained from the Research Ethics Committee of the GZA Hospitals St Augustinus, the Ethics Committee of the Medical University of Vienna, and the National Scientific and Ethics Committee of Hungary.

### Staining of tissue sections, histopathological analysis, and preclinical models

Details of the procedures that were used for tissue staining, scoring of histopathological growth patterns, scoring of breast cancer subtypes (which were determined as per published guidelines 25, 26, 27) and *in vivo* models can be found in supplementary material, Supplementary materials and methods. The Institute of Cancer Research Animal Ethics Committee granted approval for animal work, and procedures were performed in accordance with the UK Home Office regulations.

### Statistical analysis

Statistical analysis was performed with a two‐tailed Fisher's exact test or a two‐tailed Student's *t*‐test. *P*‐values of <0.05 were considered to be significant.

## Results

### Human lung metastases present with distinct growth patterns that are associated with different vascularization mechanisms

To investigate the mechanisms of tumour vascularization in human lung metastases, we performed a histopathological analysis on 164 human lung metastasis cases (46 breast cancer metastases, 57 colorectal cancer metastases, and 61 renal cancer metastases). We identified four distinct histopathological growth patterns (HGPs): the alveolar HGP, the interstitial HGP, the perivascular cuffing HGP, and the pushing HGP. We present evidence that, although pushing HGP lung metastases utilize angiogenesis, tumour vascularization occurs through vessel co‐option in the alveolar, interstitial and perivascular cuffing HGPs.

### Co‐option of alveolar capillaries in the alveolar HGP

**Figure 1:**
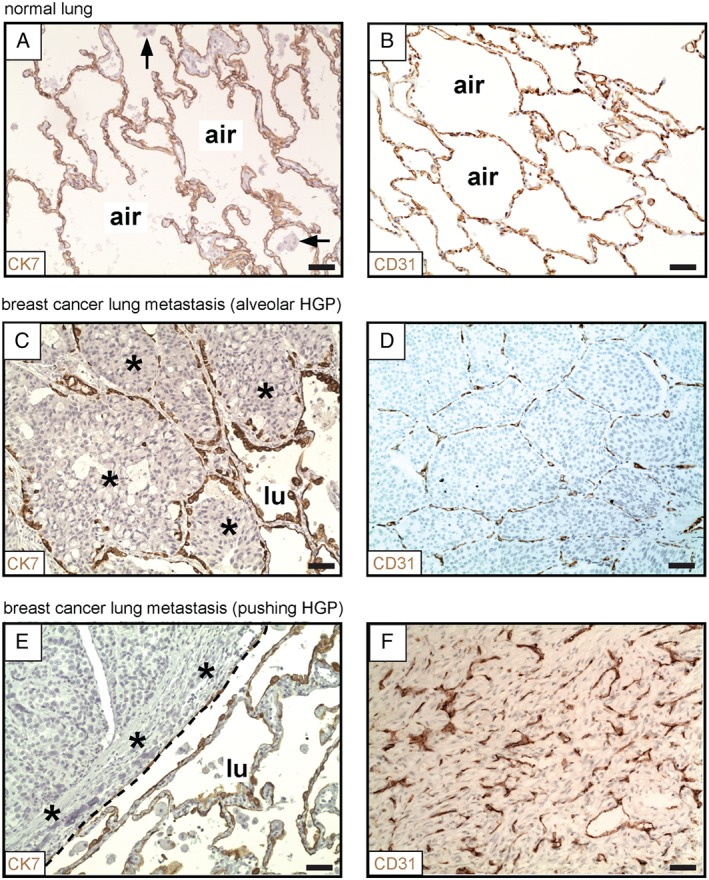
Alveolar and pushing growth patterns of human lung metastases. (A, B) Normal human lung parenchyma, stained for CK7 (A) or CD31 (B). (C, D) Alveolar HGP of human breast cancer lung metastasis. Staining for CK7 (at the tumour–lung interface) is shown in (C). Staining for CD31 (within the tumour) is shown in (D). (E, F) Pushing HGP of human breast cancer lung metastasis. Staining for CK7 (at the tumour–lung interface) is shown in (E). Staining for CD31 (within the tumour) is shown in (F). Cancer cells: asterisks. Alveolar macrophages: arrows. Alveolar air space: air. Tumour–lung interface: dashed line. Normal lung: lu. Scale bar: 50 µm.

In previous studies, incorporation of intact alveolar walls into lung metastases has been cited as evidence that these tumours co‐opt pre‐existing alveolar capillaries 13, 14, 16. In order to robustly identify the presence of alveolar walls in human tissue specimens, we performed staining for the established pneumocyte marker cytokeratin (CK) 7 28. In normal human lung, CK7 staining demonstrated a network of CK7‐positive alveolar walls separated by intervening alveolar air spaces (Figure 1A). This ‘honeycomb’ morphology is characteristic of normal human lung parenchyma. Staining for a second established pneumocyte marker, thyroid transcription factor 1 29, gave similar results.

We then performed staining for pneumocytes in samples of human breast cancer lung metastases. This approach clearly demonstrated two of the growth patterns of human lung metastases: the alveolar HGP and the pushing HGP. In the alveolar HGP, the cancer cells at the periphery of the metastasis entered the alveolar air spaces of the lung, which led to the incorporation of intact alveolar walls into the tumour (Figure 1C). In contrast, in the pushing HGP, the cancer cells did not enter the alveolar air spaces. Instead, the alveolar walls at the periphery of the metastases were pushed away by the tumour (Figure 1E).

Additionally, we examined blood vessels by staining for the vascular endothelial marker CD31. In normal human lung, a honeycomb network of CD31‐positive alveolar walls, separated by intervening alveolar air spaces, was observed (Figure 1B). Importantly, in metastases with an alveolar HGP, the tumour vessel architecture closely resembled the vascular architecture of the normal lung (Figure 1D), suggesting that these tumours do co‐opt pre‐existing alveolar capillaries by growing within the alveolar air spaces 8, 9, 10, 13. However, in sharp contrast, pushing HGP metastases contained abnormal and chaotically organized vessels (Figure 1F), which is typical of vessels generated by tumour angiogenesis 8, 9, 10, 13. Equivalent growth patterns were observed in both colorectal cancer and renal cancer lung metastases (supplementary material, Figures S4 and S5). Our interpretation of these data is that, whereas alveolar HGP lung metastases utilize vessel co‐option to obtain a vascular supply, pushing HGP lung metastases utilize angiogenesis.

**Figure 2:**
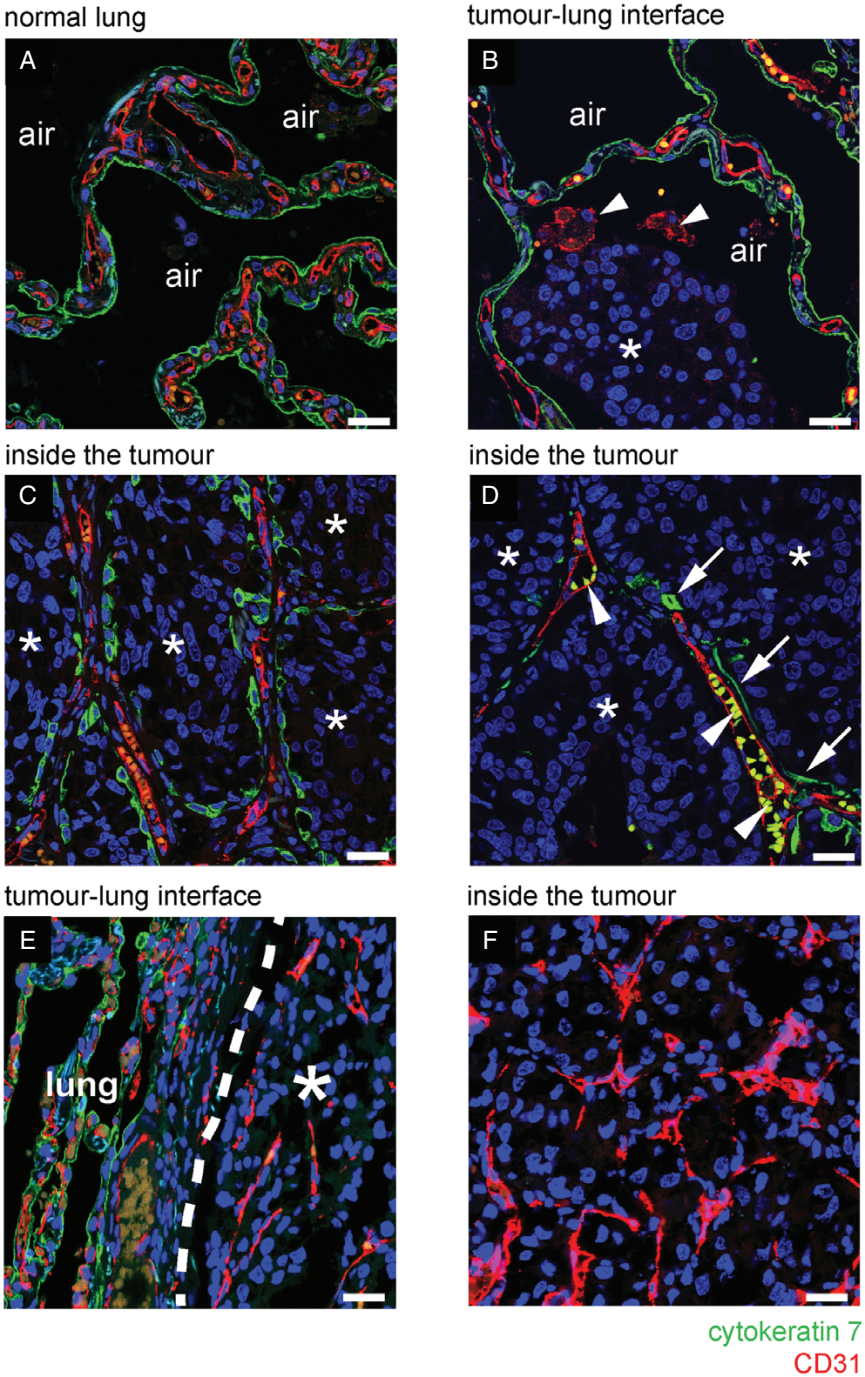
Vessel co‐option occurs in the alveolar growth pattern of human lung metastases. (A–D) Immunofluorescence co‐staining for CD31 (red) and CK7 (green) in a case of human breast cancer lung metastasis that presented with an alveolar HGP. (A) In areas of tumour‐free normal lung parenchyma, the alveolar walls are composed of CD31‐positive alveolar capillaries (red) that are sheathed by CK7‐positive pneumocytes (green). (B) At the tumour–lung interface, cancer cells (asterisks) invade an alveolar air space. Arrowheads indicate two CD31‐positive alveolar macrophages in the alveolar air space that also reacted with the CD31 antibody. (C) Behind the tumour–lung interface, cancer cells (asterisks) completely fill the alveolar air spaces, preserving the alveolar walls and the associated alveolar capillaries. (D) Towards the centre of the metastatic lesion, co‐opted alveolar capillaries can be found that are only partially coated by pneumocytes. Arrows indicate pneumocytes that are still associated with co‐opted alveolar capillaries. Arrowheads indicate autofluorescent erythrocytes in the lumen of co‐opted alveolar capillaries. (E, F) Immunofluorescence co‐staining for CD31 (red) and CK7 (green) in a sample of human renal cancer lung metastasis with a pushing HGP. At the tumour–lung interface, cancer cells push the alveolar walls away (E). No incorporation of alveolar walls was observed either at the tumour–lung interface (E) or deeper into the metastasis (F). Alveolar air space: air. Normal lung: lung. Scale bar: 25 µm.

To further characterize the mechanism of vessel co‐option, lung metastases were co‐stained for CK7 and CD31. In normal lung, this staining demonstrated the architecture of the normal alveolar walls, which are lined by CK7‐positive pneumocytes and contain CD31‐positive alveolar capillaries (Figure 2A). At the tumour–lung interface of alveolar HGP metastases, cancer cells invaded into the air spaces, facilitating the co‐option of CD31/CK7‐positive alveolar walls into the metastases (Figure 2B). Just behind the tumour–lung interface, the alveolar air spaces were fully occupied by cancer cells, but the co‐opted CD31/CK7‐positive alveolar walls remained intact (Figure 2C; supplementary material, Figure S6A–C). Co‐opted alveolar capillaries often contained erythrocytes, confirming that the co‐opted blood vessels were perfused (supplementary material, Figure S6D, E).

Moving towards the centre of the metastases, CD31‐positive alveolar capillaries could be found that were now only partially associated with CK7‐positive pneumocytes, suggesting that alveolar epithelium is gradually lost from co‐opted alveolar capillaries (Figure 2D). To further corroborate this, alveolar HGP lung metastases stained for CK7 were viewed at low power. Whereas pneumocyte‐rich alveolar walls were incorporated at the periphery of the metastases, a gradual loss of pneumocytes towards the centre of the metastases was evident (supplementary material, Figure S7). Our interpretation of these data is that cancer cells first co‐opt alveolar walls by invading the alveolar air spaces and that, subsequently, pneumocytes are gradually lost from these co‐opted alveolar walls. However, after loss of these pneumocytes, the co‐opted alveolar capillaries are retained by the tumour. In contrast, in pushing HGP lung metastases, we found no incorporation of alveolar walls into the tumour, suggesting that pushing HGP lung metastases do not co‐opt alveolar capillaries (Figure 2E, F).

### Co‐option of alveolar capillaries in the interstitial HGP

**Figure 3:**
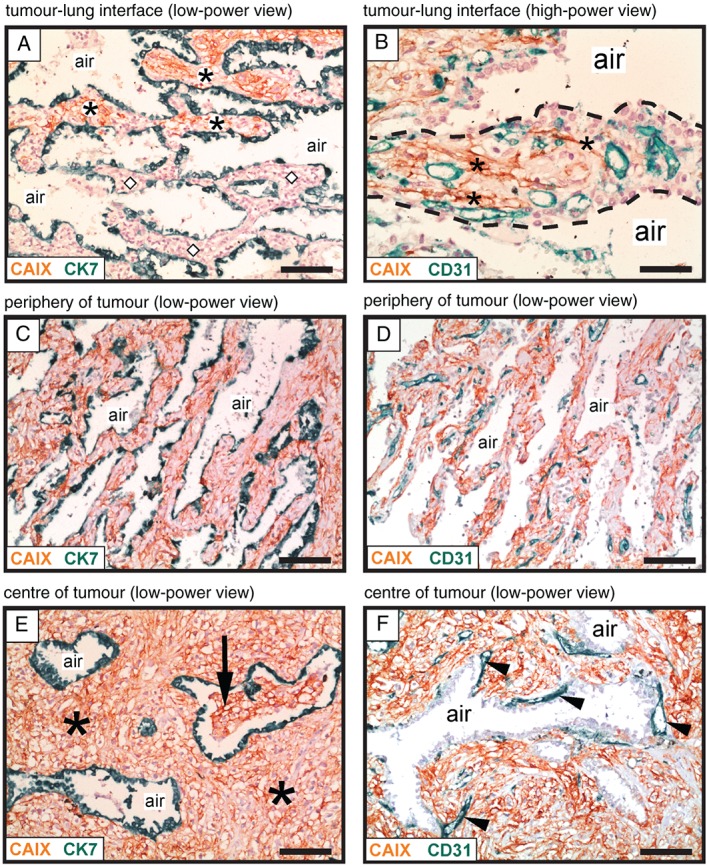
Vessel co‐option occurs in the interstitial growth pattern of human lung metastases. Immunohistochemical analysis of a renal cancer lung metastasis with an interstitial HGP, illustrating growth of cancer cells within the alveolar walls. Staining for CAIX (brown) was used to detect cancer cells, in combination with either CK7 staining (green) to detect pneumocytes (A, C, E) or CD31 staining (green) to detect blood vessels (B, D, F). (A) Tumour–lung interface: alveolar walls filled with cancer cells are present at the top of the image (asterisks), whereas tumour‐free alveolar walls of the normal lung are present below (diamond symbols). (B) High‐power view of an alveolar wall (delineated with a dashed line). Asterisks indicate cancer cells that are infiltrating around pre‐existing alveolar capillaries. (C, D) The area just behind the tumour–lung interface: the alveolar walls are now completely filled with cancer cells. The intervening alveolar air spaces remain intact. (E, F) The centre of the metastasis. In (E), asterisks indicate cancer cells that are filling the expanded alveolar walls, while the intervening alveolar air spaces remain intact. The arrow indicates an alveolar air space that has become partially filled with cancer cells. In (F), arrowheads indicate blood vessels that are closely associated with the abluminal side of an alveolar air space. Alveolar air space: air. Scale bars: 100 µm (A, C, D, E, F) and 50 µm (B).

In this study, we also observed a second growth pattern via which human lung metastases may co‐opt alveolar capillaries. In the interstitial HGP, cancer cells were seen to co‐opt alveolar capillaries by growing within the alveolar walls of the lung. The interstitial HGP is illustrated here with a case of renal cancer lung metastasis (Figure 3). Co‐staining for carbonic anhydrase 9 (CAIX), to detect renal cancer cells, and CK7, to detect pneumocytes, demonstrated the infiltration of cancer cells into the normal alveolar walls at the tumour–lung interface (Figure 3A). To verify that this mode of infiltrative growth permits co‐option of alveolar capillaries, co‐staining for CAIX and CD31 was utilized. Close inspection of the alveolar walls at the tumour–lung interface demonstrated thin columns of cancer cells invading through the alveolar interstitium between the pre‐existing alveolar capillaries (Figure 3B). Our interpretation of these data is that cancer cells can also invade through the alveolar walls to facilitate the co‐option of alveolar capillaries.

Just behind the tumour–lung interface, CAIX‐positive cancer cells completely filled the alveolar walls, but the intervening alveolar air spaces were preserved (Figure 3C). Importantly, these cancer‐filled alveolar walls contained an abundance of co‐opted alveolar capillaries (Figure 3D). Deeper into the metastasis, expansion of the cancer cell population resulted in significant broadening of the alveolar walls, but the intervening alveolar air spaces were mostly still preserved (Figure 3E). Some invasion of cancer cells into the alveolar air spaces was, however, also detected in the centre of the metastasis (Figure 3E). Within the metastasis, we often observed blood vessels that were closely associated with the abluminal side of the air spaces (Figure 3F). If our interpretation of the data is correct, the close association of these particular vessels with pneumocytes, deep within the metastasis, indicates that these are co‐opted alveolar capillaries rather than newly formed vessels. However, we cannot completely rule out the possibility that angiogenesis also occurs in this growth pattern, especially in the centre of the metastasis. We named this growth pattern the interstitial HGP because of the propensity for cancer cells to grow within the alveolar interstitium, and to be consistent with a previous report of a similar growth pattern 14.

### Co‐option of large blood vessels in the perivascular cuffing HGP

Thus far, we have described two mechanisms via which cancer cells co‐opt alveolar capillaries. However, the lungs also contain larger vessels (i.e. arteries and veins) that are distinct from alveolar capillaries because of their larger calibre and because they are surrounded by a layer of smooth muscle cells (the tunica media) (supplementary material, Figure 8A, B). In the perivascular cuffing HGP of human lung metastases, the cancer cells grow exclusively like a cuff around these larger vessels (supplementary material, Figure 8C–F). The cuff can be several layers of cancer cells thick, but is devoid of additional blood vessels. Our interpretation of these data is that the cancer cells utilize the central co‐opted vessel as their principal vascular supply in this growth pattern.

### Frequency of the different growth patterns in human lung metastases

**Figure 4:**
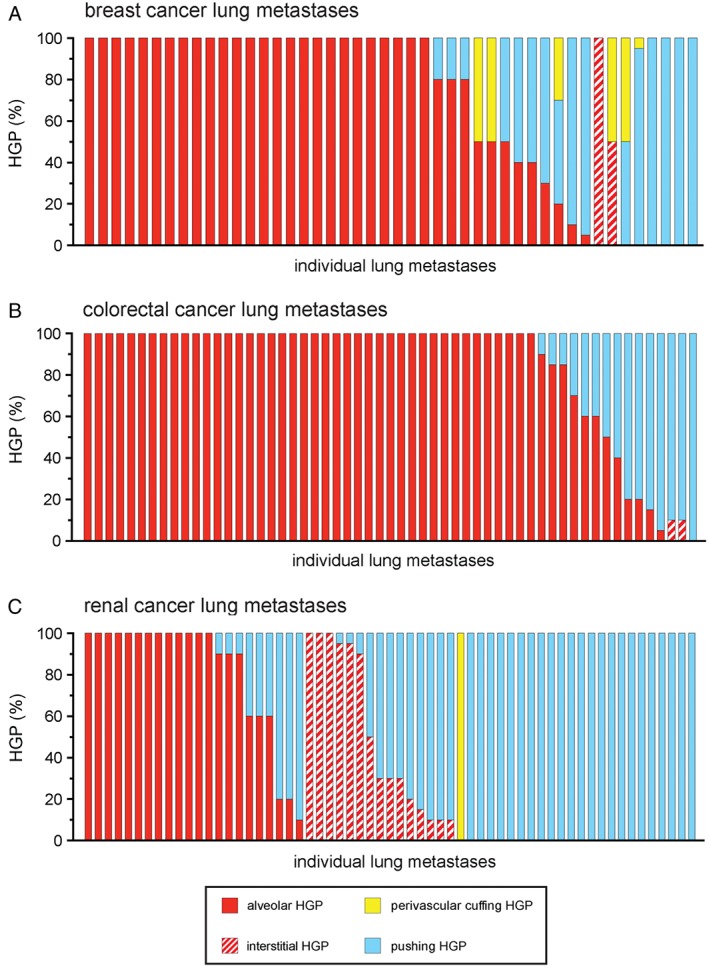
Frequency of the different HGPs in lung metastases of human breast, colorectal and renal cancer. Lung metastases of human breast cancer (A), human colorectal cancer (B) and human renal cancer (C) were scored for their growth pattern. Each bar represents an individual case of metastasis showing the percentage of the tumour–lung interface scored as alveolar, interstitial, perivascular cuffing or pushing HGP. n = 46 breast cancer lung metastases (A), n = 57 colorectal cancer lung metastases (B), and n = 61 renal cancer lung metastases (C).

To evaluate the frequencies of the alveolar, interstitial, perivascular cuffing and pushing HGPs in human lung metastases, all 164 cases were scored for their HGP (Figure 4A–C). It is of note that some lesions presented with more than one growth pattern. Therefore, the percentage of the tumour–lung interface adopting each growth pattern was scored in intervals of 5%.

To formally analyse the prevalence of vessel co‐option across the different tumour types, growth patterns that utilize vessel co‐option (alveolar, interstitial, and perivascular cuffing) were pooled together, and their incidence was compared with the incidence of the angiogenic pushing growth pattern. Vessel co‐opting growth patterns were present to some extent (≥5% of the tumour–lung interface) in 91.3% of breast, 98.2% of colorectal and 62.3% of renal cancer metastases. Moreover, vessel co‐opting growth patterns were dominant (≥75% of the tumour–lung interface) in 71.7% of breast, 78.9% of colorectal and 37.7% of renal cancer metastases. Vessel co‐opting growth patterns were more common in breast cancer than in renal cancer (*p* = 0.0008, Fisher's exact test), and in colorectal cancer than in renal cancer (*p* < 0.0001, Fisher's exact test).

Breast cancer metastases were also characterized for intrinsic molecular subtype: luminal A, luminal B (HER2‐negative), luminal B (HER2‐positive), HER2‐positive (non‐luminal), and triple‐negative. Vessel co‐opting growth patterns were present across all subtypes (supplementary material, Figure S9). However, vessel co‐option was less prevalent in triple‐negative tumours than in other subtypes (*p* = 0.022, Fisher's exact test).

### Limited efficacy of sunitinib in lung metastasis models as compared with subcutaneously implanted tumours

Conventional anti‐angiogenic therapies were designed to inhibit new blood vessel growth, but were not designed to target vessel co‐option. To investigate whether vessel co‐option could mediate resistance to anti‐angiogenic therapy in lung metastases, we utilized three preclinical syngeneic tumour models corresponding to the three types of human cancer studied above. The 4 T1 cell line was used to model breast cancer, and the C26 and RENCA cell lines were used to model colorectal and renal cancer, respectively. To examine the response to anti‐angiogenic therapy, we utilized the potent anti‐angiogenic tyrosine kinase inhibitor sunitinib.

**Figure 5:**
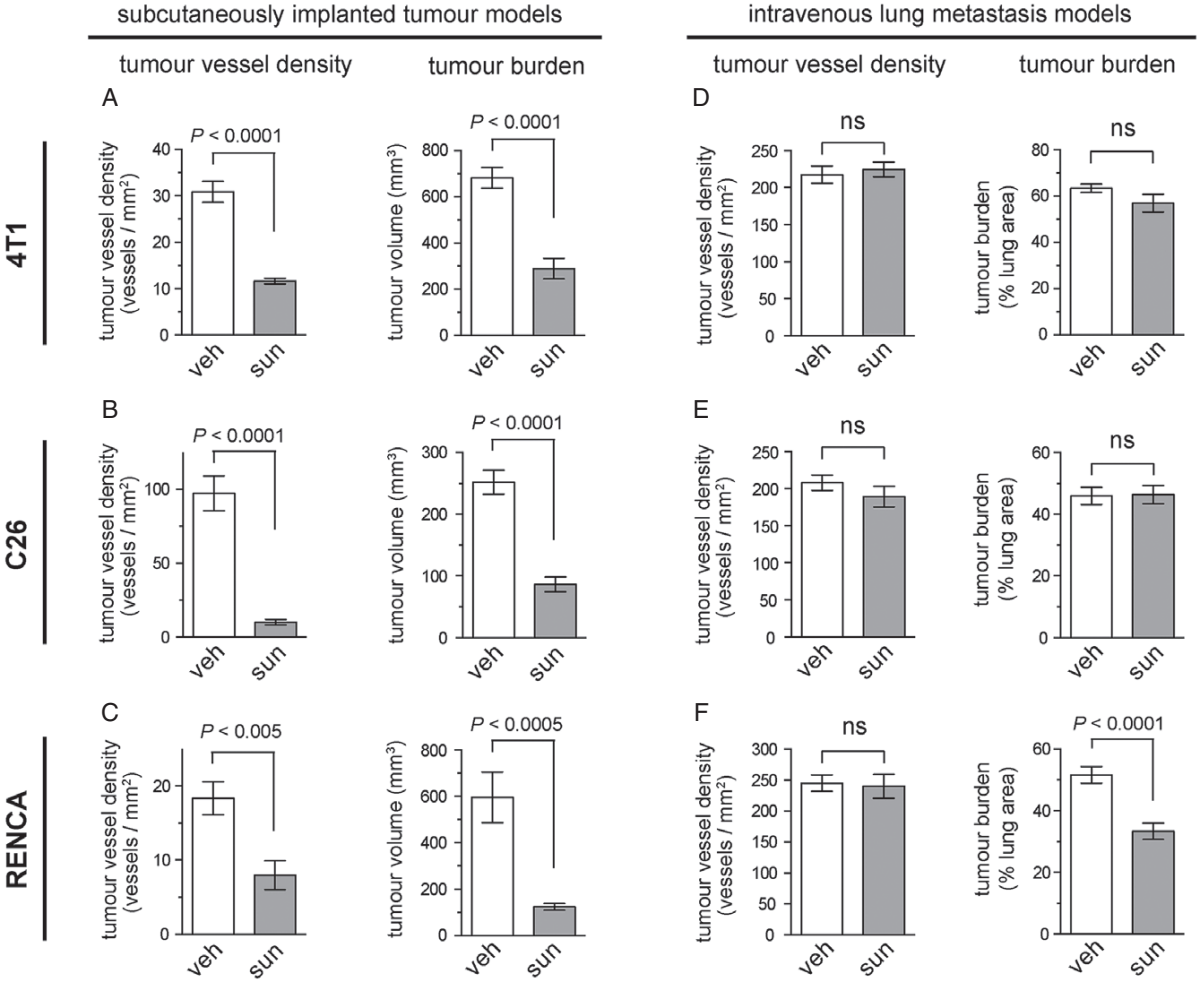
Limited efficacy of sunitinib in lung metastasis models as compared with subcutaneously implanted tumours. (A–C) The efficacy of sunitinib was tested in mice injected subcutaneously with 4 T1 (A), C26 (B) or RENCA (C) cells. The graphs show tumour vessel density ± standard error of the mean (SEM) (left) or tumour burden ± SEM (right) in subcutaneous 4 T1 (A), C26 (B) or RENCA (C) tumours after 10 days of treatment with either 40 mg/kg per day sunitinib or vehicle alone. n = 10 mice per experimental group for tumour burden graphs. n = 6 mice per experimental group for tumour vessel density graphs. (D–F) Mice were injected via the tail vein with 4 T1 (D), C26 (E) or RENCA (F) cells. The graphs show tumour vessel density ± SEM (left) or tumour burden ± SEM (right) in the lungs after 10 days of treatment with either 40 mg/kg per day sunitinib or vehicle alone. n = 9 or 10 mice per experimental group for tumour burden graphs. n = 5 mice per experimental group for tumour vessel density graphs. ns, no significant difference; sun, sunitinib; veh, vehicle.

As the growth of subcutaneously implanted tumours is known to be angiogenesis‐dependent, we first assessed sunitinib activity against subcutaneously implanted 4 T1, C26 and RENCA tumours. Treatment with 40 mg/kg per day sunitinib for 10 days significantly suppressed both tumour vessel density and tumour burden in all three models (Figure 5A–C), confirming the ability of sunitinib to suppress tumour angiogenesis and tumour growth in these three angiogenesis‐dependent tumour models.

We then investigated the response to sunitinib in lung metastases formed by the same cell lines. Lung metastases were established by intravenous tail vein injection of 4 T1, C26 or RENCA cells. In contrast to its potent activity against subcutaneously implanted tumours, the same sunitinib treatment regimen (40 mg/kg per day sunitinib for 10 days) did not significantly suppress tumour vessel density in any of the lung metastasis models (Figure 5D–F). In addition, sunitinib treatment did not reduce tumour burden in either 4 T1 or C26 lung metastases (Figure 5D, E). Sunitinib treatment did significantly suppress tumour burden by ∼34% in RENCA lung metastases (Figure 5F). However, this activity is modest when compared with subcutaneously implanted RENCA tumours, in which the same treatment regimen suppressed tumour burden by ∼80% (Figure 5C).

### Evidence that vessel co‐option mediates resistance to sunitinib in 4 T1 and C26 lung metastases

**Figure 6:**
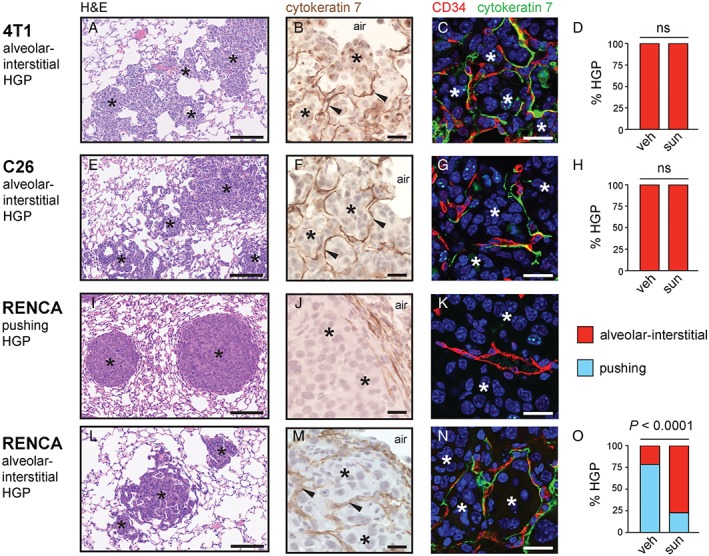
Evidence for vessel co‐option in lung metastasis models. Histopathological characterization was performed on lung metastases formed by 4 T1 cells (A–D), C26 cells (E–H) and RENCA cells (I–O) after tail vein injection. (A, E, I, L) Low‐power views of lung metastasis morphology by H&E staining. (B, C, E, F, I, J, L, M) Higher‐power views of CK7‐stained lung metastases (B, F, J, M) or lung metastases co‐stained for CD34 (red) and CK7 (green) (C, G, K, N). The graphs show percentage alveolar/interstitial HGP and percentage pushing HGP scored in 4 T1 (D), C26 (H) and RENCA (O) lung metastases from vehicle (veh)‐treated or sunitinib (sun)‐treated mice (n = 9 or 10 mice per experimental group). Cancer cells: asterisks. Alveolar air spaces: air. ns, no significant difference. Scale bars: 125 µm (A, E, I, L), 20 µm (B, F, J, M) and 20 µm (C, G, K, N).

To determine why the lung metastases responded so poorly to this anti‐angiogenic drug, we examined the histopathological growth patterns of all three lung metastasis models (Figure 6). 4 T1 and C26 lung metastases had irregular margins and were highly infiltrative into the lung parenchyma (Figure 6A, E). Closer examination revealed that the cancer cells colonized the lung by growing in the alveolar air spaces and/or by growing in the alveolar walls (supplementary material, Figure S10). Staining for the pneumocyte marker CK7 revealed that the alveolar walls of the lung were clearly incorporated into the metastases (Figure 6B, F). Moreover, co‐staining for CK7 and the blood vessel marker CD34 highlighted the presence of blood vessels in these tumours that were still associated with alveolar epithelial cells, showing that these tumours incorporate alveolar capillaries (Figure 6C, G). Therefore, the 4 T1 and C26 models have a growth pattern that mimics the alveolar/interstitial HGP of human lung metastases, and these tumours co‐opt pre‐existing alveolar capillaries. Quantification of the growth pattern in 4 T1 and C26 lung metastases revealed that this alveolar/interstitial HGP was the dominant growth pattern in both the vehicle‐treated and sunitinib‐treated mice (Figure 6D, H).

Our interpretation of these data is that the 4 T1 and C26 models of lung metastasis co‐opt pre‐existing alveolar capillaries, and that these co‐opted vessels are not sensitive to sunitinib treatment. These data provide a potential mechanistic explanation for the inability of sunitinib to control tumour burden in these lung metastasis models.

### Evidence that a switch from angiogenesis to vessel co‐option mediates resistance to sunitinib in RENCA lung metastases

The situation was more complex for RENCA lung metastases, which presented with a mixture of pushing HGP metastases (Figure 6I–K) and alveolar/interstitial HGP metastases (Figure 6L–N). In vehicle‐treated mice, the pushing HGP was the prevalent growth pattern of RENCA lung metastases (Figure 6O). These pushing HGP metastases had a strikingly spherical or ‘cannonball’ shape (Figure 6I), and, instead of invading the lung parenchyma, these tumours pushed the alveolar walls away (Figure 6J). The blood vessels in pushing HGP metastases were not associated with CK7‐positive pneumocytes, suggesting that these tumours vascularize through angiogenesis instead of vessel co‐option (Figure 6K).

However, in sunitinib‐treated mice, the alveolar/interstitial HGP was the prevalent growth pattern of RENCA lung metastases (Figure 6O). RENCA lung metastases with an alveolar/interstitial HGP had an irregular margin and infiltrated the lung parenchyma (Figure 6L). Staining for CK7 revealed incorporation of alveolar walls into the metastases (Figure 6M), and co‐staining for CD34 and CK7 highlighted that these tumours incorporate pre‐existing alveolar capillaries (Figure 6N).

In the analysis of vessel density in RENCA lung metastases described above (Figure 5F), vessel density was quantified across the entire tissue section without regard to the growth pattern. We therefore re‐examined vessel density in RENCA lung metastases by quantifying, separately, the vessel density in pushing HGP lesions and the vessel density in alveolar/interstitial HGP lesions. Importantly, whereas vessel density was significantly lower in pushing HGP RENCA lesions from sunitinib‐treated mice than in the vehicle group (supplementary material, Figure S11A–C), no significant difference in vessel density was observed between alveolar/interstitial HGP RENCA lesions from sunitinib‐treated mice and the vehicle group (supplementary material, Figure S11D–F).

Our interpretation of these data is that: 1 the vessels of angiogenic pushing growth pattern RENCA lung metastases are sensitive to sunitinib; and 2 sunitinib also induces a rapid shift in growth pattern to the alveolar/interstitial HGP, which permits the co‐option of alveolar capillaries that are resistant to sunitinib. This provides a potential mechanistic explanation for why sunitinib has only a modest effect on tumour burden in the RENCA lung metastasis model.

## Discussion

Here, we examined the mechanisms of tumour vascularization in human lung metastases. We report three distinct growth patterns of human lung metastases in which, if our interpretation of the histology is correct, the co‐option of pre‐existing vessels occurs via three distinct mechanisms. In the alveolar HGP, cancer cells invade the alveolar air spaces, which facilitates the co‐option of the alveolar capillaries that lie within the incorporated alveolar walls. In the interstitial HGP, cancer cells infiltrate the alveolar walls, which again allows the co‐option of alveolar capillaries, albeit via a mechanism that is distinct from that in the alveolar HGP. In the perivascular cuffing HGP, cancer cells grow as a cuff around large pre‐existing vessels of the lung, resulting in the co‐option of these larger vessels. We also report a pushing HGP in which the lung metastases present with a chaotically organized vasculature that is typical of tumour angiogenesis. Our interpretation of these data is that lung metastases can vascularize by co‐opting pre‐existing vessels of the lung (via three distinct mechanisms) or can utilize angiogenesis. Moreover, rather than being a rare event, we found that vessel co‐option occurs frequently in human lung metastases.

We do acknowledge, however, that both angiogenesis and vessel co‐option can occur within the same lesion. In support of this, we observed here that lung metastases can present with a mixture of growth patterns e.g. lesions in which the pushing HGP and the alveolar HGP were both present, but in different areas of the same lesion. Moreover, in both the alveolar HGP and the interstitial HGP, it is possible that new vessels can sprout from co‐opted vessels once the co‐opted vessels are in the centre of the metastasis. In support of this, studies on NSCLC have shown that many of these tumours can invade the alveolar air spaces at the tumour periphery (permitting vessel co‐option), but that a switch to angiogenesis then occurs in the centre of the tumour 8, 9. Presumably, this occurs because vessels co‐opted at the periphery are induced to undergo angiogenesis when they find themselves within the centre of the tumour. We therefore propose that there is both spatial heterogeneity and temporal heterogeneity in the vascularization mechanisms used by human tumours, with lesions being able to utilize either angiogenesis or vessel co‐option or both.

It is not currently clear why cancer cells utilize vessel co‐option instead of, or as well as, activating angiogenesis when they metastasize to the lung. Previous work has suggested that, although micrometastases can rely on vessel co‐option, tumours must switch to a reliance on angiogenesis as they become larger 30. However, in the current study, we observed vessel co‐option even in human lung metastases that were large (≥1 cm in diameter). If we interpret these data correctly, we must conclude that vessel co‐option can be a mechanism of tumour vascularization in both micrometastases and macrometastases. In addition, our data suggest that tumours of diverse primary origin (i.e. breast, bowel, and kidney) can all utilize vessel co‐option when they metastasize to lung. Given this evidence, it seems probable that the environment of the lung plays an active role in inducing cancer cells to utilize vessel co‐option instead of angiogenesis.

We also provide evidence that vessel co‐option mediates intrinsic resistance to anti‐angiogenic therapy in preclinical lung metastasis models. Whereas the anti‐angiogenic drug sunitinib suppressed the growth of angiogenesis‐dependent subcutaneously implanted 4 T1 and C26 tumours, the lung metastases of 4 T1 and C26 tumours utilized vessel co‐option and showed intrinsic resistance to this same anti‐angiogenic drug. Further evidence that vessel co‐option mediates intrinsic resistance to anti‐angiogenic therapy comes from a spontaneous breast cancer metastasis model using the highly metastatic MDA‐MB‐231^LM2–4^ breast cancer cell line. We reported previously that, whereas the growth of MDA‐MB‐231^LM2–4^ tumours is significantly suppressed by sunitinib when these cells are implanted orthotopically in the mammary fat pad, administration of sunitinib does not prolong the survival of mice bearing spontaneous MDA‐MB‐231^LM2–4^ metastases 31. Importantly, we found that, whereas mammary fat pad‐implanted MDA‐MB‐231^LM2–4^ tumours are angiogenic, spontaneous MDA‐MB‐231^LM2–4^ lung metastases utilize vessel co‐option (Harold Dvorak and Robert Kerbel, unpublished observation). In confirmation of this, staining for CK7 and CD34 in spontaneous MDA‐MB‐231^LM2–4^ lung metastases demonstrated that these tumours have an alveolar HGP and that they do co‐opt alveolar capillaries (supplementary material, Figure S12). If our interpretation of these data is correct, vessel co‐option is therefore associated with intrinsic resistance to sunitinib, not just in intravenous models of lung metastasis (i.e. 4 T1 and C26), but also in a model of spontaneous breast cancer lung metastasis using a human breast cancer cell line (i.e. MDA‐MB‐231^LM2–4^).

Vessel co‐option might also mediate acquired resistance to anti‐angiogenic therapy. Although RENCA lung metastases presented mainly as angiogenic pushing HGP lesions in vehicle‐treated mice, sunitinib induced a switch to an alveolar/interstitial HGP that vascularizes by vessel co‐option. Importantly, whereas the vessels of pushing HGP metastases were sensitive to sunitinib, the vessels of alveolar/interstitial HGP lung metastases were not. Our interpretation of these data is that a treatment‐induced switch from angiogenesis to vessel co‐option could drive acquired resistance to anti‐angiogenic therapy. In further support of this, Kuczynski *et al* recently showed that a switch from angiogenesis to vessel co‐option drives acquired resistance to the anti‐angiogenic drug sorafenib in hepatocellular carcinoma 20. Moreover, a switch from angiogenesis to vessel co‐option has been reported to occur in some brain malignancies treated with anti‐angiogenic therapy 17, 18, 19, 23, 24.

Four phase 3 trials have tested sunitinib in metastatic breast cancer, with no benefit in either progression‐free survival (PFS) or overall survival (OS) being demonstrated for sunitinib 32, 33, 34, 35. If the quantification of vessel co‐option in human breast cancer lung metastases presented here is representative of breast cancer patients as a whole, many patients entering these trials will have presented with breast cancer metastases to the lung that vascularize through vessel co‐option. In addition, vessel co‐option occurs in breast cancer metastases to the skin 36, lymph nodes 21, 37, liver 22, 38, and brain 39, 40, 41. Therefore, vessel co‐option may help to explain, at least in part, why anti‐angiogenic therapy has been a disappointing therapeutic approach in metastatic breast cancer.

In contrast to its effect on breast cancer, sunitinib extends both PFS and OS in metastatic renal cancer 42, 43. Here, we found that vessel co‐option occurs less frequently in human renal cancer lung metastases than in human breast cancer lung metastases. This disparity may help to explain why sunitinib is a clinically more effective treatment for metastatic renal cancer than for metastatic breast cancer. Nonetheless, both intrinsic and acquired resistance to anti‐angiogenic drugs occur in renal cancer patients 44, 45. Moreover, despite showing activity in the advanced disease setting, sunitinib did not prolong disease‐free survival in renal cancer patients who received this drug as adjuvant therapy 46. We propose that vessel co‐option may help to explain resistance to anti‐angiogenic therapy of renal cancer in both the metastatic setting and the adjuvant setting.

In conclusion, if we have interpreted our data correctly, vessel co‐option is a common event in human lung metastases that occurs via three distinct mechanisms. Moreover, our data suggest that vessel co‐option may drive resistance to anti‐angiogenic therapy in preclinical lung metastasis models. One limitation of our study is that, owing to a lack of sufficient lung metastasis samples from patients treated with anti‐angiogenic therapy, we have not been able to examine whether there is an association between vessel co‐option and resistance to anti‐angiogenic therapy in patients. There are, however, clinical data showing that co‐option of the pre‐existing vasculature is associated with resistance to anti‐angiogenic therapy in both lymph node metastases 21 and liver metastases 22. Further research is therefore now warranted to confirm the role of vessel co‐option in resistance to anti‐angiogenic therapy in patients with lung metastases.

If vessel co‐option does prove to be a common mechanism of resistance to anti‐angiogenic therapy across multiple metastatic sites, then therapeutic approaches that can inhibit both angiogenesis and vessel co‐option may be warranted for the treatment of metastatic disease. However, further research will now be required to establish how both modalities of tumour vascularization can be effectively targeted simultaneously in patients.

### Author contributions statement

The authors contributed in the following way: VLB, PBV, SF, FD, EK, MRN, EW, SF, ARR: performed the experiments, collected the data, and analysed the data; VLB, PBV, AB, TS, BH, KH, FR, BD, ARR: organized the collection of tissue samples and the associated clinical data; PBV, EAK, NSV, JL, MG, HFD, SP, RSK, BD: assisted with interpretation of the data and provided critical comments on the manuscript; VLB, PBV, SP, BD, ARR: conceived of and designed the study. VLB, ARR: prepared the figures and wrote the manuscript. All authors gave final approval to the submitted version of the paper.

**SUPPLEMENTARY MATERIAL ONLINE**

**Supplementary materials and methods**

**Supplementary figure legends**

**Figure S1.** Consort diagram for breast cancer lung metastasis cases.

**Figure S2.** Consort diagram for colorectal cancer lung metastasis cases.

**Figure S3.** Consort diagram for renal cancer lung metastasis cases.

**Figure S4.** Alveolar HGP and pushing HGP of colorectal cancer lung metastases.

**Figure S5.** Alveolar HGP and pushing HGP of renal cancer lung metastases.

**Figure S6.** Examples of co‐opted alveolar capillaries in human lung metastases.

**Figure S7.** Pattern of pneumocyte staining in the alveolar HGP.

**Figure S8.** Vessel co‐option in the perivascular cuffing growth pattern of human lung metastases.

**Figure S9.** Cases of breast cancer lung metastases grouped by intrinsic molecular subtype.

**Figure S10.** High power views of alveolar growth pattern and interstitial growth pattern in preclinical lung metastasis models.

**Figure S11.** Contrasting effect of sunitinib in RENCA lung metastases with different HGPs.

**Figure S12.** Vessel co‐option in spontaneous MDA‐MB‐231^LM2–4^ lung metastases.

**Table S1.** Clinical characteristics of breast cancer patients.

**Table S2.** Clinical characteristics of colorectal cancer patients.

**Table S3.** Clinical characteristics of renal cancer patients.

## Supporting information


**Supplementary materials and methods**

**Supplementary figure legends**

**Figure S1. Consort diagram for breast cancer lung metastasis cases** Consort diagram to demonstrate the selection of breast cancer lung metastasis cases for this study. Where samples were excluded, the reasons for exclusion are indicated.

**Figure S2. Consort diagram for colorectal cancer lung metastasis cases** Consort diagram to demonstrate the selection of colorectal cancer lung metastasis cases for this study. Where samples were excluded, the reasons for exclusion are indicated.

**Figure S3. Consort diagram for renal cancer lung metastasis cases** Consort diagram to demonstrate the selection of renal cancer lung metastasis cases for this study. Where samples were excluded, the reasons for exclusion are indicated.

**Figure S4. Alveolar HGP and pushing HGP of colorectal cancer lung metastases A,B.** Alveolar HGP of colorectal cancer lung metastasis. Panel A shows tumour‐lung interface stained for cytokeratin 7 (CK7). Note the presence of cancer cells (asterisks) within the alveolar air spaces. Panel B shows an intra‐tumoural region stained for CD31. Note that the vascular architecture of the tumour mimics the vascular architecture of normal lung parenchyma (see Figure 1B for CD31 staining of normal lung for comparison). **C,D.** Pushing HGP of colorectal cancer lung metastasis. Panel C shows tumour‐lung interface stained for cytokeratin 7 (CK7). Note that the cancer cells (asterisks) push the alveolar walls away. Panel D shows an intra‐tumoural region stained for CD31. Note that the vasculature is chaotic which is typical for the process of tumour angiogenesis. Cancer cells (asterisk), normal lung (lu). Scale bars, 50 µm.

**Figure S5. Alveolar HGP and pushing HGP of renal cancer lung metastases A,B.** Alveolar HGP of renal cancer lung metastasis. Panel A shows tumour‐lung interface stained for cytokeratin 7 (CK7). Note the presence of cancer cells (asterisks) within the alveolar air spaces. Panel B shows an intra‐tumoural region stained for CD31. Note that the vascular architecture of the tumour mimics the vascular architecture of normal lung parenchyma (see Figure 1B for CD31 staining of normal lung for comparison). **C,D.** Pushing HGP of renal cancer lung metastasis. Panel C shows tumour‐lung interface stained for cytokeratin 7 (CK7). Note that the cancer cells (asterisks) push the alveolar walls away. Panel D shows an intra‐tumoural region stained for CD31. Note that the vasculature is chaotic which is typical for the process of tumour angiogenesis. Cancer cells (asterisk), normal lung (lu). Scale bars, 50 µm.

**Figure S6. Examples of co‐opted alveolar capillaries in human lung metastases A‐C.** Immunofluorescence co‐staining for CD31 (red) and cytokeratin 7 (CK7, green) in human lung metastases presenting with an alveolar HGP. Examples of co‐opted alveolar capillaries are shown from lung metastases of human breast cancer (**A**), human colorectal cancer (**B**) and human renal cancer (**C**). **D,E.** Serial sections from a case of human breast cancer lung metastasis with an alveolar HGP were stained for H&E (**D**) or cytokeratin 7 (CK7) (**E**). Arrows point to erythrocytes within a co‐opted alveolar capillary, indicating that the co‐opted vessel is perfused and functional. Cancer cells (asterisks), co‐opted alveolar capillaries (arrowheads), erythrocytes (arrows). Scale bars, 25 µm.

**Figure S7. Pattern of pneumocyte staining in the alveolar HGP** Lower power view of a human breast cancer lung metastasis, which presented with an alveolar HGP. The case has been stained for the pneumocyte marker cytokeratin 7 (CK7). Three zones are indicated: zone 1 (normal lung), zone 2 (periphery of the metastasis where the alveolar epithelium is mostly preserved within the metastasis) and zone 3 (centre of the metastasis where the alveolar epithelium begins to fragment). Dotted line indicates the tumour‐lung interface. Scale bar, 500 µm.

**Figure S8. Vessel co‐option in the perivascular cuffing growth pattern of human lung metastases A,B.** Images of normal human lung parenchyma stained for CD31. Arrows indicate large blood vessels. Arrowheads indicate the surrounding smooth muscle layer (tunica media). **C,D.** Images of human breast cancer lung metastasis with a perivascular‐cuffing HGP stained for CD31. The central co‐opted vessel is indicated (arrow). The cancer cells that form a cuff around the vessel are also indicated (asterisks). **E,F.** Images of a human breast cancer lung metastasis with a perivascular‐cuffing HGP which was stained for oestrogen receptor alpha (ERα) to detect the cancer cells. The ERα‐positive cancer cells (asterisks) grow as a cuff around the large vessels. Arrows indicate the large central vessels that are co‐opted. Scale bar, 50 µm.

**Figure S9. Cases of breast cancer lung metastases grouped by intrinsic molecular subtype** Graph shows the HGPs for 46 cases of breast cancer lung metastases (the same as scored in Figure 4A) grouped here by intrinsic molecular subtype. Lum A = luminal A; Lum B (HER2‐) = luminal B (HER2 negative); Lum B (HER2+) = luminal B (HER2 positive); HER2+ (non‐lum) = HER2 positive (non‐luminal) and TN = triple negative. For one of the cases we were unable to determine the subtype due to lack of sufficient tissue samples (ND, not determined).

**Figure S10. High power views of alveolar growth pattern and interstitial growth pattern in preclinical lung metastasis models A.** Alveolar growth pattern. In lung metastases formed by 4T1 cells, groups of 4T1 cells growing in alveolar air spaces are indicated with an arrowhead. **B.** Interstitial growth pattern. In lung metastases formed by C26 cells, groups of C26 cells that are growing within the alveolar walls are indicated with asterisks. Scale bar, 100 µm.

**Figure S11. Contrasting effect of sunitinib in RENCA lung metastases with different HGPs A‐F.** Graphs show tumour vessel density +/− SEM in lung metastases from mice injected via the tail vein with RENCA cells and then treated for 10 days with either 40 mg/kg/day sunitinib (sun) or vehicle (veh) alone (**A,D**). Vessel density was quantified in pushing lung metastases (**A**) or alveolar‐interstitial lung metastases (**D**) separately. n = 20 lung metastases (from 5 mice) per data point. Representative images of CD34 staining in pushing lung metastases (**B,C**) and alveolar‐interstitial lung metastases (**E,F**) from vehicle (**B,E**) or sunitinib (**C,F**) treated mice are shown. No significant difference (ns).

**Figure S12. Vessel co‐option in spontaneous MDA‐MB‐231^LM2–4^ lung metastases A,B.** Staining for CD34, to demonstrate blood vessels (red), and cytokeratin 7 (CK7), to demonstrate pneumocytes (green), in normal mouse lung (**A**) or spontaneous lung metastases of MDA‐MB‐231^LM2–4^ cells (**B**). Note that the alveolar structure of the normal lung (**A**) is preserved within the lung metastases (**B**) indicating that the lung metastases in this model grow with an alveolar HGP and co‐opt pre‐existing alveolar capillaries. Asterisks indicate breast cancer cells present in the alveolar air spaces. Arrows indicate alveolar macrophages. Scale bar, 20 μM.

**Table S1. Clinical characteristics of breast cancer patients** Clinical characteristics of 46 patients with breast cancer lung metastases that were included in the study.
**Table S2. Clinical characteristics of colorectal cancer patients** Clinical characteristics of 53 patients with colorectal cancer lung metastases that were included in the study.
**Table S3. Clinical characteristics of renal cancer patients** Clinical characteristics of 59 patients with renal cancer lung metastases that were included in the study.
