# Supplementary material for: Vessel co‐option is common in human lung metastases and mediates resistance to anti‐angiogenic therapy in preclinical lung metastasis models
Source: J Pathol. 2016 Dec 29;241(3):362–74. doi: 10.1002/path.4845 (PMC5248628; doi:10.1002/path.4845)
Supplement: Supplementary file 1 — Supplementary materials and methods [file PATH-241-362-s001.docx]

**Supplementary materials and methods**

**Details of primary antibodies**

The primary antibodies used were: 1:1000 anti-carbonic anhydrase IX (CAIX) (ab15086, Abcam, Cambridge, UK), 1:30 anti-CD31 (M0823, Dako, Ely, UK), 1:100 anti-CD34 (ab8188, Abcam), 1:50 anti-cytokeratin 7 (CK7) (sc23876, Santa Cruz, Dallas, TX), 1:80 anti-oestrogen receptor alpha (ERα) (M3643, Dako), 1:300 anti-Ki67 (M7240, Dako), 1:200 anti-progesterone receptor (PgR) (M 3569, Dako) and 1:50 anti-thyroid transcription factor 1 (TTF1) (M3575, Dako).

**Staining of tissue sections**

For staining of human and mouse tissue, 4 μm sections from FFPE blocks were de-paraffinised and rehydrated by standard protocols. Antigen retrieval was performed either at pH 6 in a pressure cooker or at pH 9 in a microwave. Sections were incubated for 1 h at room temperature or overnight at 4 °C with primary antibodies diluted in REAL diluent (DAKO). For immunofluorescence, primary antibodies were detected with Alexa-488 or Alexa-555 fluorescently-conjugated secondary antibodies (Invitrogen) diluted in REAL diluent supplemented with DAPI for 30 mins at room temperature, followed by mounting under glass coverslips in MOWIOL mountant supplemented with anti-fade (0.1% w/v 1,4-diazabicyclo[2,2,2]octane) (Sigma). For DAB and tetramethylbenzidine (TMB) staining, primary antibodies were detected with Envision Flex system (K8002, Dako), followed by a light counterstain with haematoxylin before mounting under glass coverslips in DPEX mountant. HER2 was detected using the HercepTest kit (SK001, Dako). Images were captured using a confocal laser-scanning microscope (Leica) or a light microscope (Olympus), as appropriate.

**Scoring of histopathological growth patterns in human lung metastasis specimens**

All lung metastasis cases underwent four different stainings which were used to score the histopathological growth pattern (HGP): (a) H&E stain, (b) immunohistochemistry for a pneumocyte marker (usually CK7, but TTF1 was also used for breast cancer), (c) immunohistochemistry for a blood vessel marker (CD31) and (d) immunofluorescence co-staining for a pneumocyte marker (CK7 or TTF1) and CD31. All specimens were digitally scanned using a semi-automated scanning microscope (Hamamatsu Nanozoomer) and HGPs were scored by reviewing the images using NDPI viewer software (Hamamatsu). Metastases were scored as having an alveolar, interstitial, perivascular cuffing or pushing HGP according to the following criteria. Alveolar HGP: the metastasis presented with an intra-alveolar growth pattern, where cancer cells invaded into the alveolar air spaces at the tumour-lung interface and there was incorporation of intact alveolar walls (and their associated capillaries) into the metastasis. Interstitial HGP: cancer cells invaded into the alveolar walls at the tumour-lung interface, facilitating the incorporation of the associated capillaries into the metastasis. Incorporation of intact alveolar air spaces into the metastasis was always clearly visible in the interstitial HGP. Perivascular cuffing HGP: cancer cells grew as a cuff around large pre-existing vessels of the lung. Pushing HGP: the cancer cells pushed the lung parenchyma away at the periphery of the metastasis (leading to compression and flattening of the adjacent alveolar walls) and the tumour vasculature did not resemble the vasculature of the normal lung parenchyma. To account for lesions presenting with a mixture of different HGPs, the percentage of the tumour-lung interface adopting the alveolar, interstitial, perivascular cuffing or pushing HGP was scored in intervals of 5% for each metastasis.

In the alveolar HGP, it was sometimes observed that cancers cells also invaded into the alveolar walls after those walls were incorporated into the metastasis. Such cases were scored as having an alveolar HGP when the intra-alveolar growth pattern was predominant at the tumour-lung interface. In the interstitial HGP, it was sometimes observed that cancer cells could invade into the air spaces after those air spaces were incorporated into the metastasis. Such cases were scored as having an interstitial HGP when the interstitial growth pattern was predominant at the tumour-lung interface.

**Scoring intrinsic subtypes of breast cancer in lung metastasis samples**

Cases of breast cancer lung metastasis were characterized for intrinsic molecular subtype: luminal A, luminal B-HER2 negative, luminal B-HER2 positive, HER2 positive (non-luminal) and triple negative using surrogate immunohistochemical markers as recommended in recently published guidelines [23]. In brief, FFPE tissue sections were stained for ER, PgR, HER2 or Ki67 and were then scored. For both ER and PgR, positive staining in ≥1% of tumour cell nuclei was required in order for the case to be considered receptor positive [24]. For HER2, the following system was utilized: 0 or 1+ (HER2 negative), 2+ (HER2 borderline), or 3+ (HER2 positive) [25]. Cases scored as HER2 borderline underwent additional testing with the HER2 CISH pharmDx kit (SK109, Dako) to test for HER2 amplification according to the manufacturer’s instructions. The presence of HER2 amplification was considered to indicate that the case was HER2 positive. Cases were deemed Ki67 ‘low’ if <14% of nuclei were Ki67 positive, otherwise they were considered to be Ki67 ‘high.’

The results of the ER, PgR, HER2 and Ki67 analyses were then used to assign each case to an intrinsic molecular subtype according to the criteria shown below and in accordance with published guidelines [23]:

| **Intrinsic subtype** | **Criteria** |
| --- | --- |
| Luminal A | ER and PgR positive  HER2 negative  Ki67 ‘low’ |
| Luminal B HER2 negative | ER positive  HER2 negative  Ki67 ‘high’ |
| Luminal B HER2 positive | ER positive  HER2 positive  Any Ki67  Any PgR |
| HER2 positive (non-luminal) | HER2 positive  ER and PgR both negative |
| Triple negative | Negative for ER, PgR and HER2 |

**Cell culture**

The murine cancer cell lines (4T1, C26 and RENCA) were cultured on plastic and maintained at 37**°**C / 5% CO_2_ in RPMI supplemented with 10% FCS. All cell lines were regularly checked for mycoplasma and shown to be contamination free.

**Preparation of sunitinib for oral dosing**

Vehicle for sunitinib consisted of 0.5 % w/v carboxymethylcellulose sodium, 1.8 % w/v NaCl, 0.4 % w/v Tween-80, 0.9 % w/v benzyl alcohol dissolved in reverse osmosis deionised water adjusted to pH 6. For oral dosing, sunitinib malate powder was added to vehicle and vortexed to create a suspension. Fresh stocks of sunitinib suspension were prepared weekly and stored at 4 °C in the dark. Oral dosing of mice was performed by administration of 0.2 ml of vehicle or sunitinib suspension by oral gavage.

**Mouse models**

The Institute of Cancer Research Animal Ethics Committee granted approval for animal work and procedures were performed in accordance with the United Kingdom Home Office regulations. Female Balb/c mice at 8 - 12 weeks of age (Charles River) were used for all *in vivo* studies. To establish subcutaneous tumours, mice were injected with 2x10^5^ 4T1 cells, 2x10^5^ C26 cells or 1x10^6^ RENCA cells. Once tumours reached a volume of 50-100 mm^2^, mice were randomised to treatment with 40 mg/kg/day sunitinib or vehicle alone. After 10 days of treatment the mice were culled and the tumours were harvested. After measurement with callipers the tumours were fixed in formalin overnight. Tumour volumes were calculated using the formula: (length x width^2^)/2. To establish lung metastases, mice were injected with 2x10^5^ 4T1, C26 or RENCA cells via the tail vein. Mice injected with 4T1 or C26 cells were randomised to treatment with sunitinib or vehicle alone on the day after injection. Due to the slower growth rate of RENCA lung metastases, mice injected with RENCA cells were randomised to treatment with sunitinib or vehicle alone at 10 days after injection. In all three models, mice were culled after 10 days of treatment. The lungs were then fixed by infusion of formalin into the lungs via the trachea, followed by immersion in formalin to fix overnight.

For quantification of tumour burden, digitally scanned H&E-stained sections of mouse lung were viewed using NDPI viewer software. The marquee tool in the software package was used freehand to measure both areas of tumour (*a*) and the total area of the lung (*b*). To calculate tumour burden, the percentage area of lung section that was occupied by tumour was then calculated according to this formula: (*a* / *b*) x 100. For quantification of vessel density in both subcutaneous tumours and lung metastases, sections were stained for CD34 and the number of CD34-positive tumour vessels was counted. Vessel density was expressed in terms of the number of vessels per mm^2^ of tumour tissue.
