# Supplementary figures and images for: Vessel co‐option is common in human lung metastases and mediates resistance to anti‐angiogenic therapy in preclinical lung metastasis models
Source: J Pathol. 2016 Dec 29;241(3):362–74. doi: 10.1002/path.4845 (PMC5248628; doi:10.1002/path.4845)

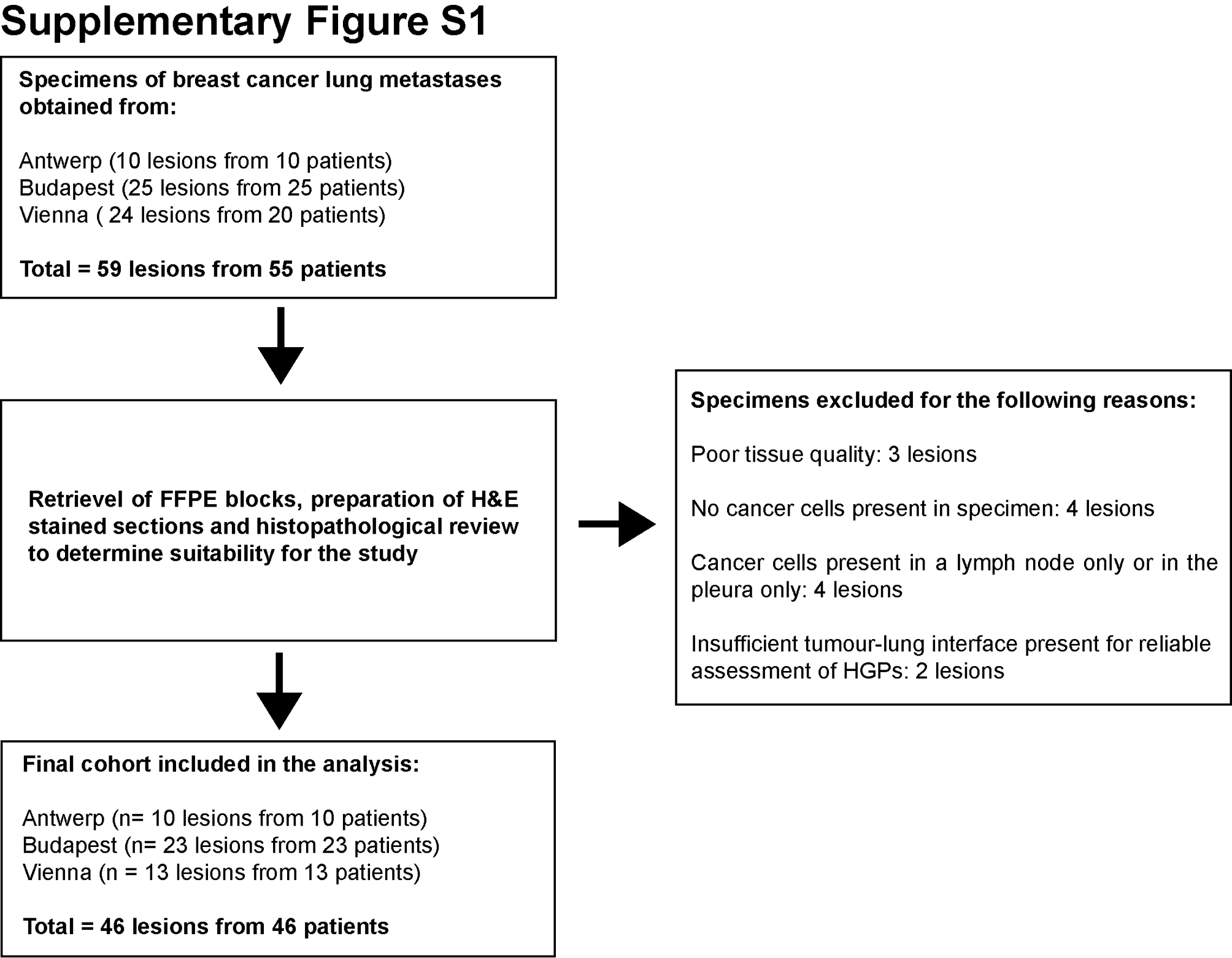

Supplement: Supplementary file 3 — Figure S1. Consort diagram for breast cancer lung metastasis cases Consort diagram to demonstrate the selection of breast cancer lung metastasis cases for this study. Where samples were excluded, the reasons for exclusion are indicated. [file PATH-241-362-s003.tif]

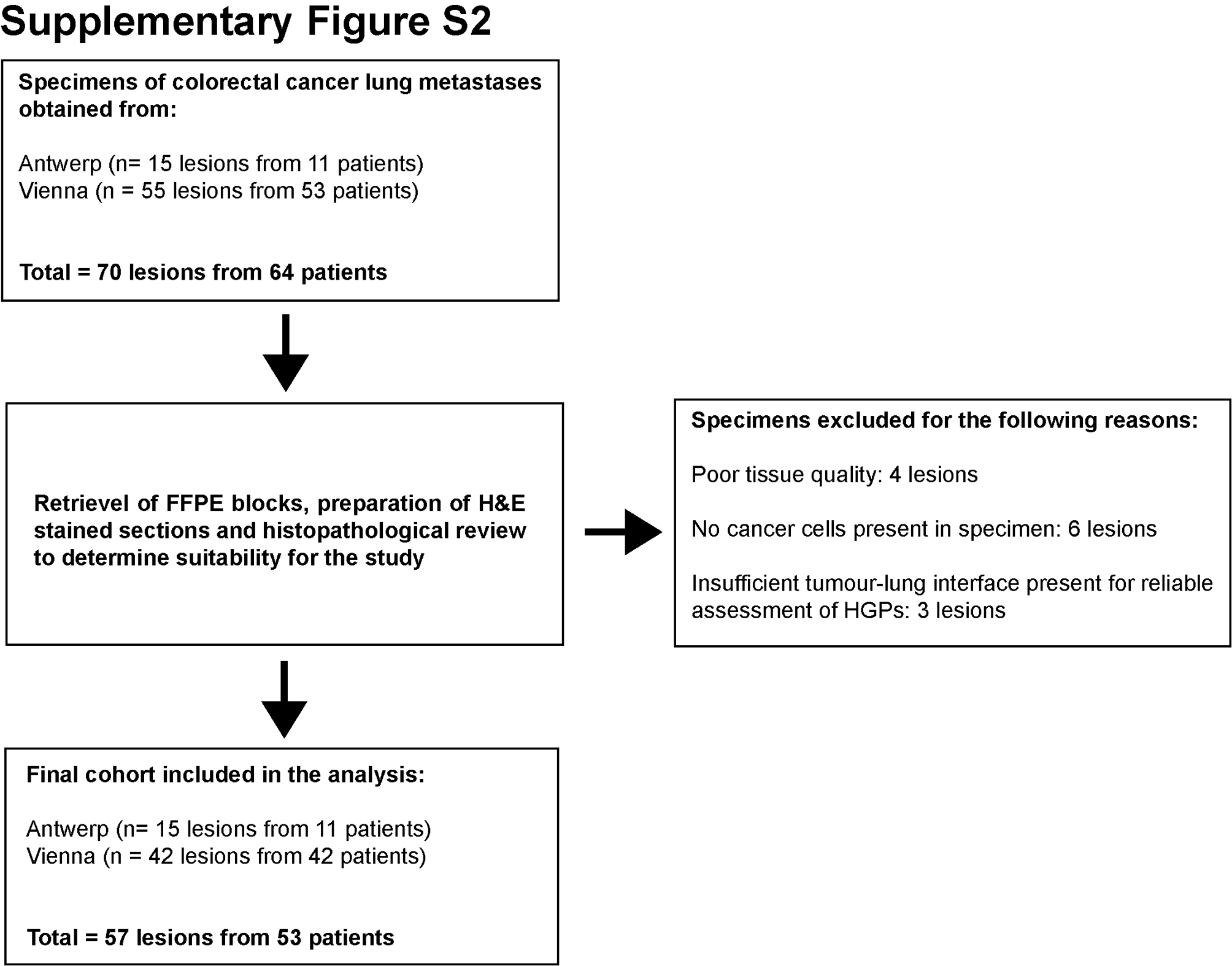

Supplement: Supplementary file 4 — Figure S2. Consort diagram for colorectal cancer lung metastasis cases Consort diagram to demonstrate the selection of colorectal cancer lung metastasis cases for this study. Where samples were excluded, the reasons for exclusion are indicated. [file PATH-241-362-s004.tif]

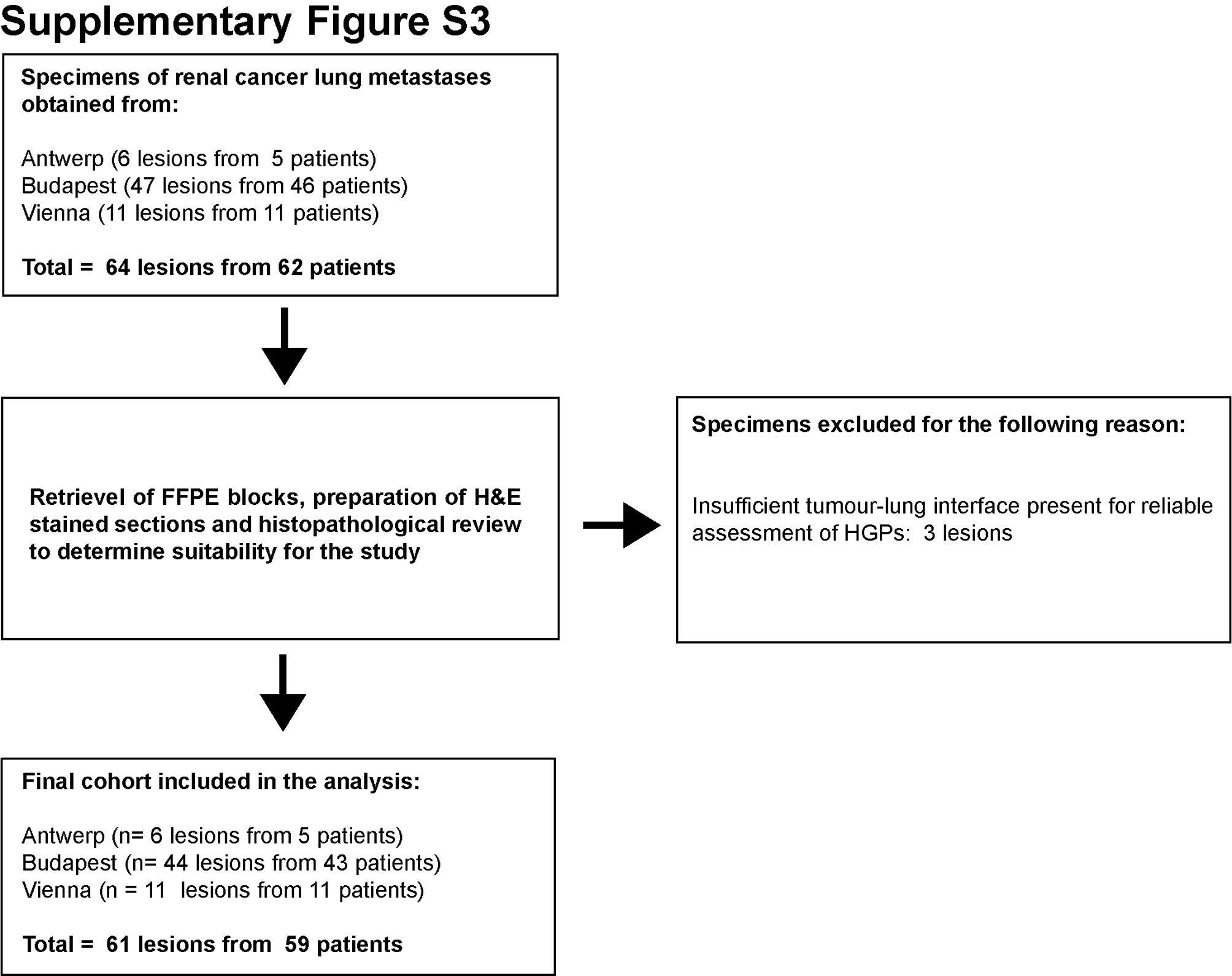

Supplement: Supplementary file 5 — Figure S3. Consort diagram for renal cancer lung metastasis cases Consort diagram to demonstrate the selection of renal cancer lung metastasis cases for this study. Where samples were excluded, the reasons for exclusion are indicated. [file PATH-241-362-s005.tif]

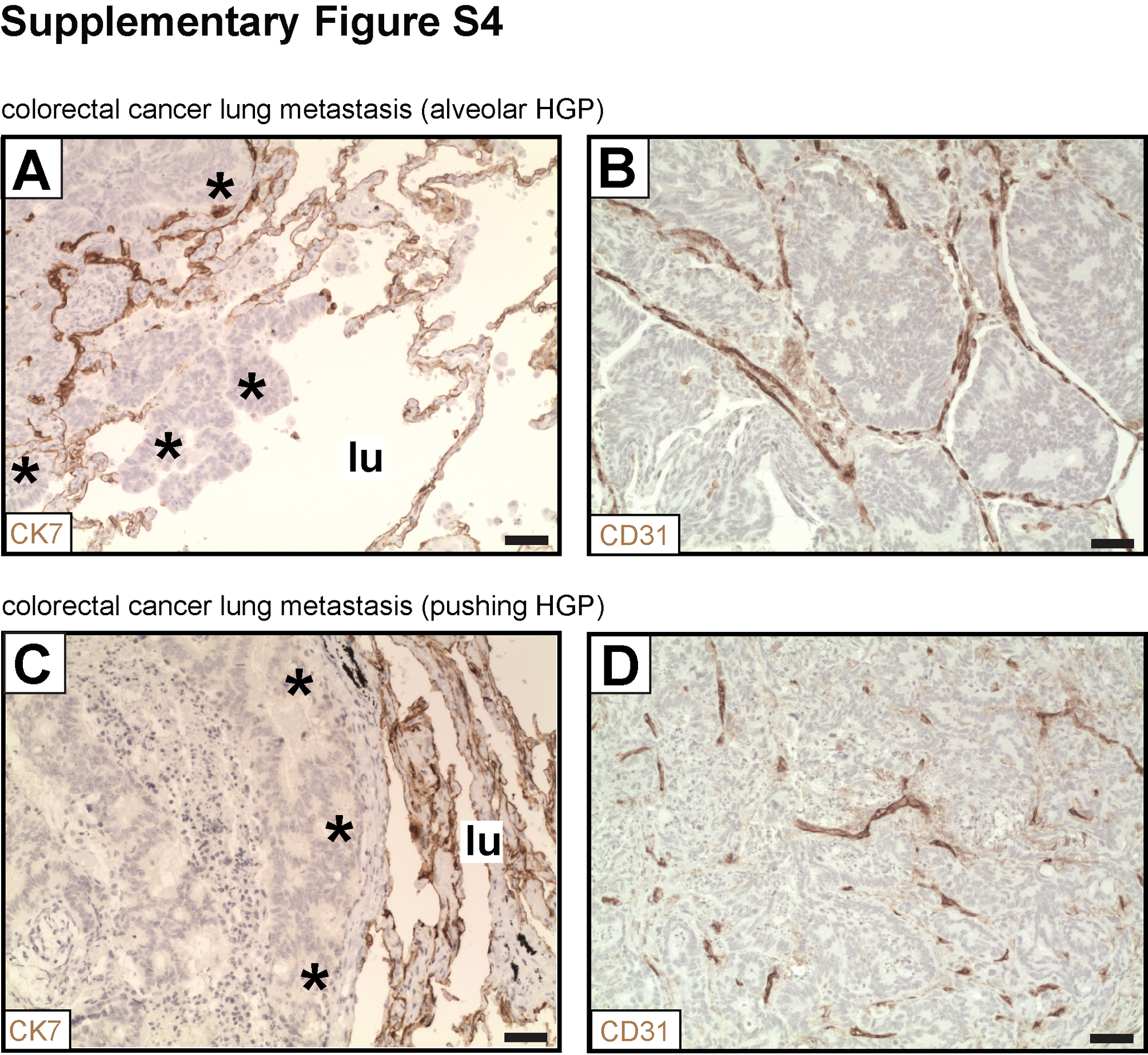

Supplement: Supplementary file 6 — Figure S4. Alveolar HGP and pushing HGP of colorectal cancer lung metastases A,B. Alveolar HGP of colorectal cancer lung metastasis. Panel A shows tumour‐lung interface stained for cytokeratin 7 (CK7). Note the presence of cancer cells (asterisks) within the alveolar air spaces. Panel B shows an intra‐tumoural region stained for CD31. Note that the vascular architecture of the tumour mimics the vascular architecture of normal lung parenchyma (see Figure 1B for CD31 staining of normal lung for comparison). C,D. Pushing HGP of colorectal cancer lung metastasis. Panel C shows tumour‐lung interface stained for cytokeratin 7 (CK7). Note that the cancer cells (asterisks) push the alveolar walls away. Panel D shows an intra‐tumoural region stained for CD31. Note that the vasculature is chaotic which is typical for the process of tumour angiogenesis. Cancer cells (asterisk), normal lung (lu). Scale bars, 50 µm. [file PATH-241-362-s006.tif]

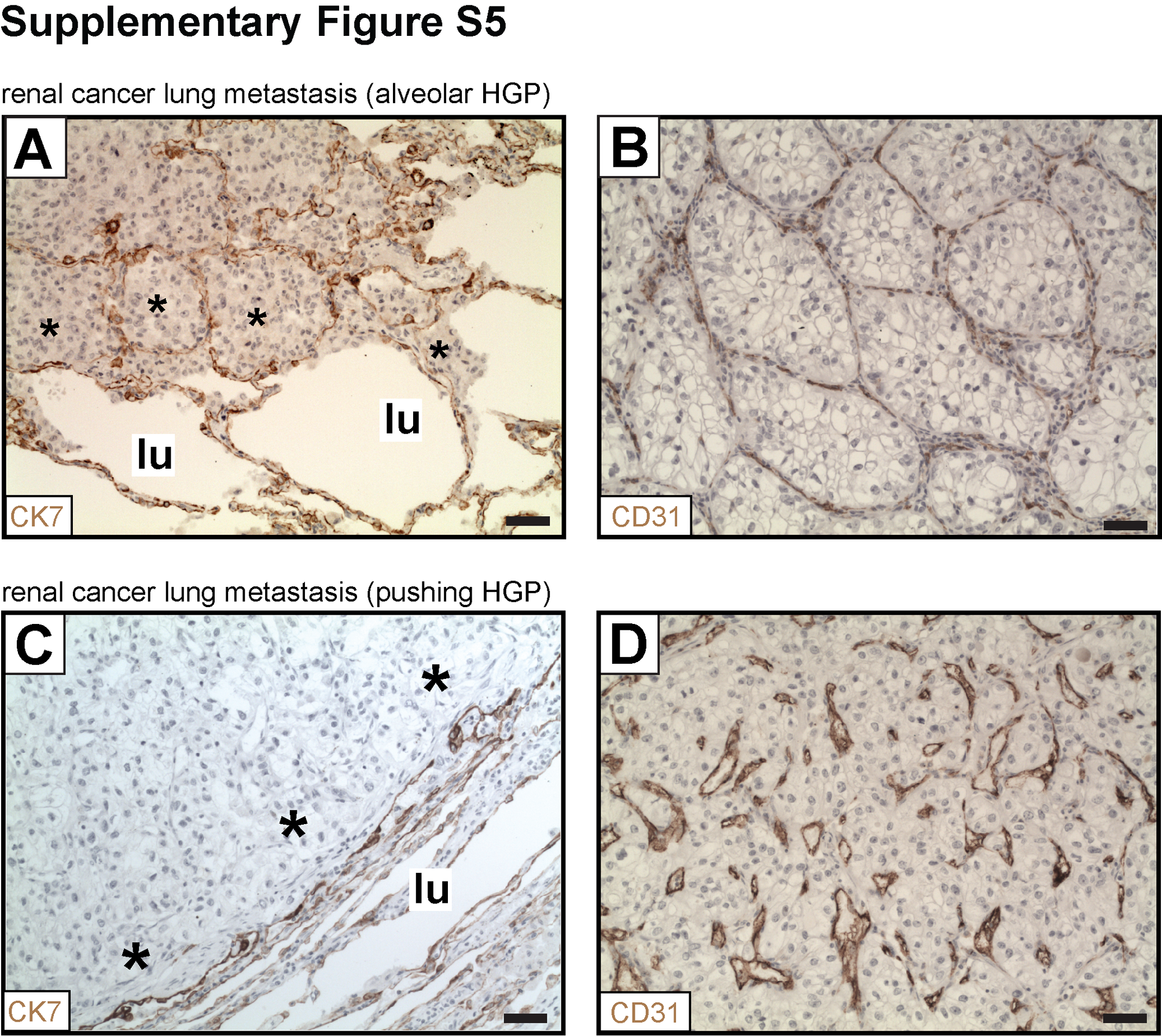

Supplement: Supplementary file 7 — Figure S5. Alveolar HGP and pushing HGP of renal cancer lung metastases A,B. Alveolar HGP of renal cancer lung metastasis. Panel A shows tumour‐lung interface stained for cytokeratin 7 (CK7). Note the presence of cancer cells (asterisks) within the alveolar air spaces. Panel B shows an intra‐tumoural region stained for CD31. Note that the vascular architecture of the tumour mimics the vascular architecture of normal lung parenchyma (see Figure 1B for CD31 staining of normal lung for comparison). C,D. Pushing HGP of renal cancer lung metastasis. Panel C shows tumour‐lung interface stained for cytokeratin 7 (CK7). Note that the cancer cells (asterisks) push the alveolar walls away. Panel D shows an intra‐tumoural region stained for CD31. Note that the vasculature is chaotic which is typical for the process of tumour angiogenesis. Cancer cells (asterisk), normal lung (lu). Scale bars, 50 µm. [file PATH-241-362-s007.tif]

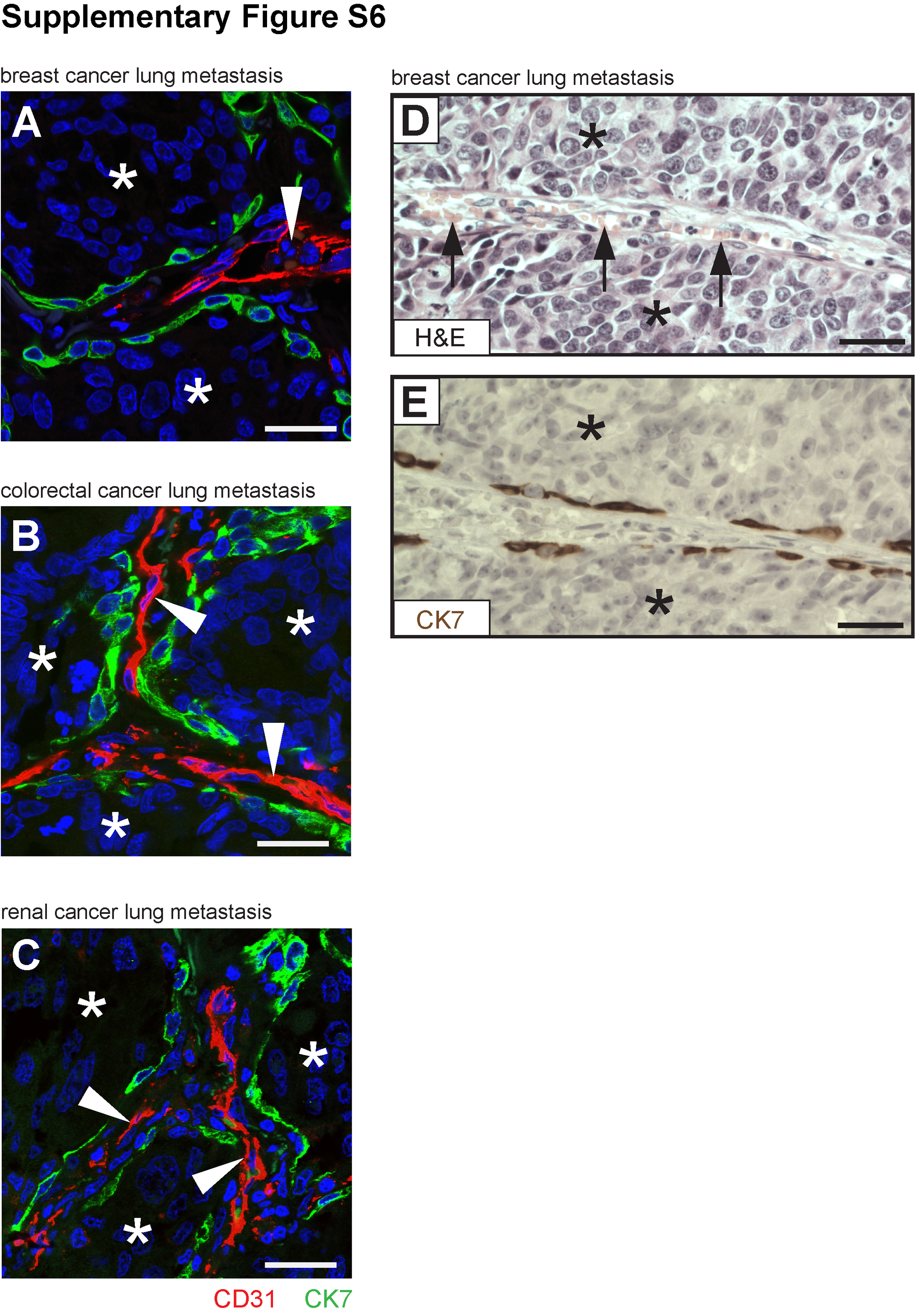

Supplement: Supplementary file 8 — Figure S6. Examples of co‐opted alveolar capillaries in human lung metastases A‐C. Immunofluorescence co‐staining for CD31 (red) and cytokeratin 7 (CK7, green) in human lung metastases presenting with an alveolar HGP. Examples of co‐opted alveolar capillaries are shown from lung metastases of human breast cancer (A), human colorectal cancer (B) and human renal cancer (C). D,E. Serial sections from a case of human breast cancer lung metastasis with an alveolar HGP were stained for H&E (D) or cytokeratin 7 (CK7) (E). Arrows point to erythrocytes within a co‐opted alveolar capillary, indicating that the co‐opted vessel is perfused and functional. Cancer cells (asterisks), co‐opted alveolar capillaries (arrowheads), erythrocytes (arrows). Scale bars, 25 µm. [file PATH-241-362-s008.tif]

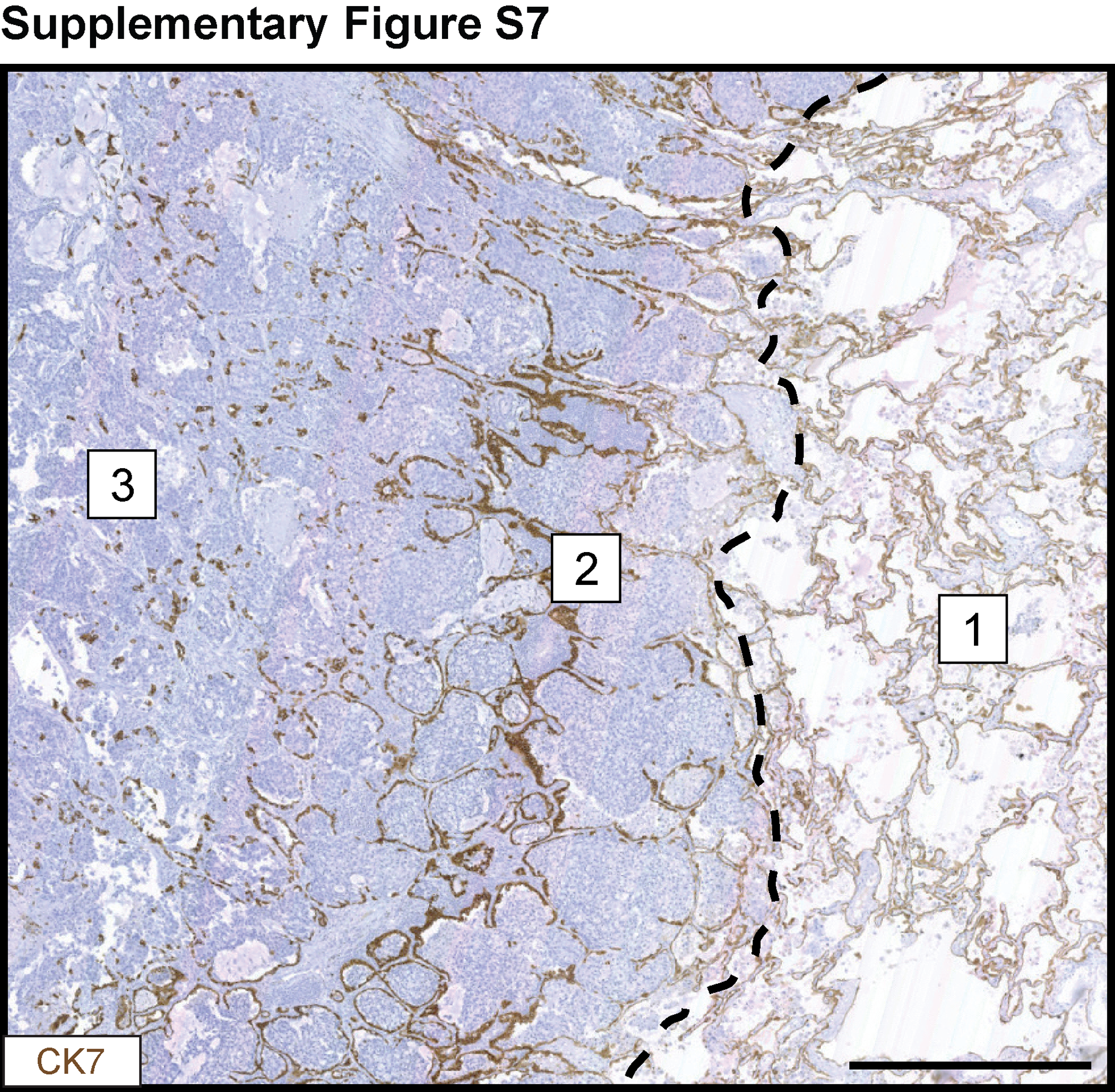

Supplement: Supplementary file 9 — Figure S7. Pattern of pneumocyte staining in the alveolar HGP Lower power view of a human breast cancer lung metastasis, which presented with an alveolar HGP. The case has been stained for the pneumocyte marker cytokeratin 7 (CK7). Three zones are indicated: zone 1 (normal lung), zone 2 (periphery of the metastasis where the alveolar epithelium is mostly preserved within the metastasis) and zone 3 (centre of the metastasis where the alveolar epithelium begins to fragment). Dotted line indicates the tumour‐lung interface. Scale bar, 500 µm. [file PATH-241-362-s009.tif]

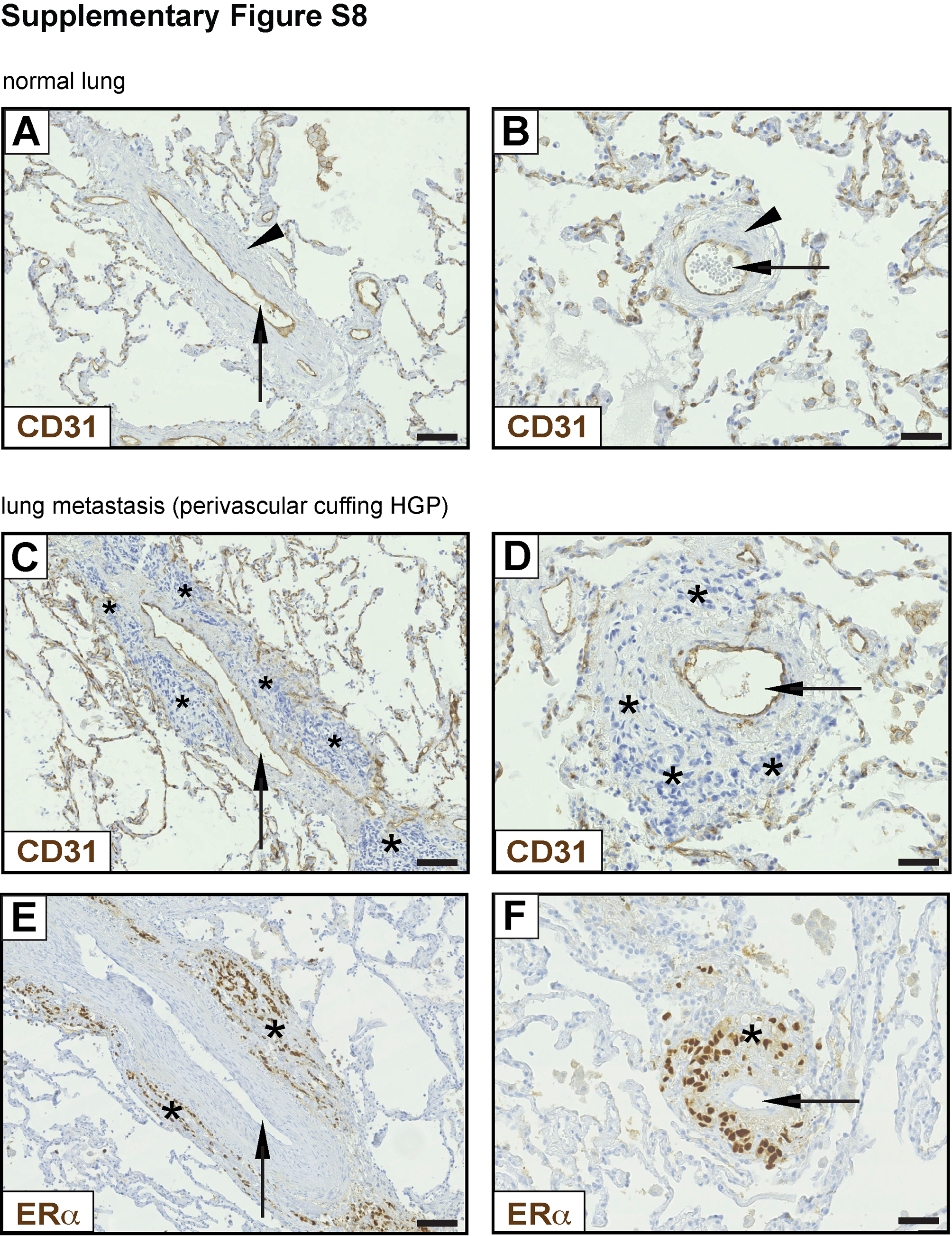

Supplement: Supplementary file 10 — Figure S8. Vessel co‐option in the perivascular cuffing growth pattern of human lung metastases A,B. Images of normal human lung parenchyma stained for CD31. Arrows indicate large blood vessels. Arrowheads indicate the surrounding smooth muscle layer (tunica media). C,D. Images of human breast cancer lung metastasis with a perivascular‐cuffing HGP stained for CD31. The central co‐opted vessel is indicated (arrow). The cancer cells that form a cuff around the vessel are also indicated (asterisks). E,F. Images of a human breast cancer lung metastasis with a perivascular‐cuffing HGP which was stained for oestrogen receptor alpha (ERα) to detect the cancer cells. The ERα‐positive cancer cells (asterisks) grow as a cuff around the large vessels. Arrows indicate the large central vessels that are co‐opted. Scale bar, 50 µm. [file PATH-241-362-s010.tif]

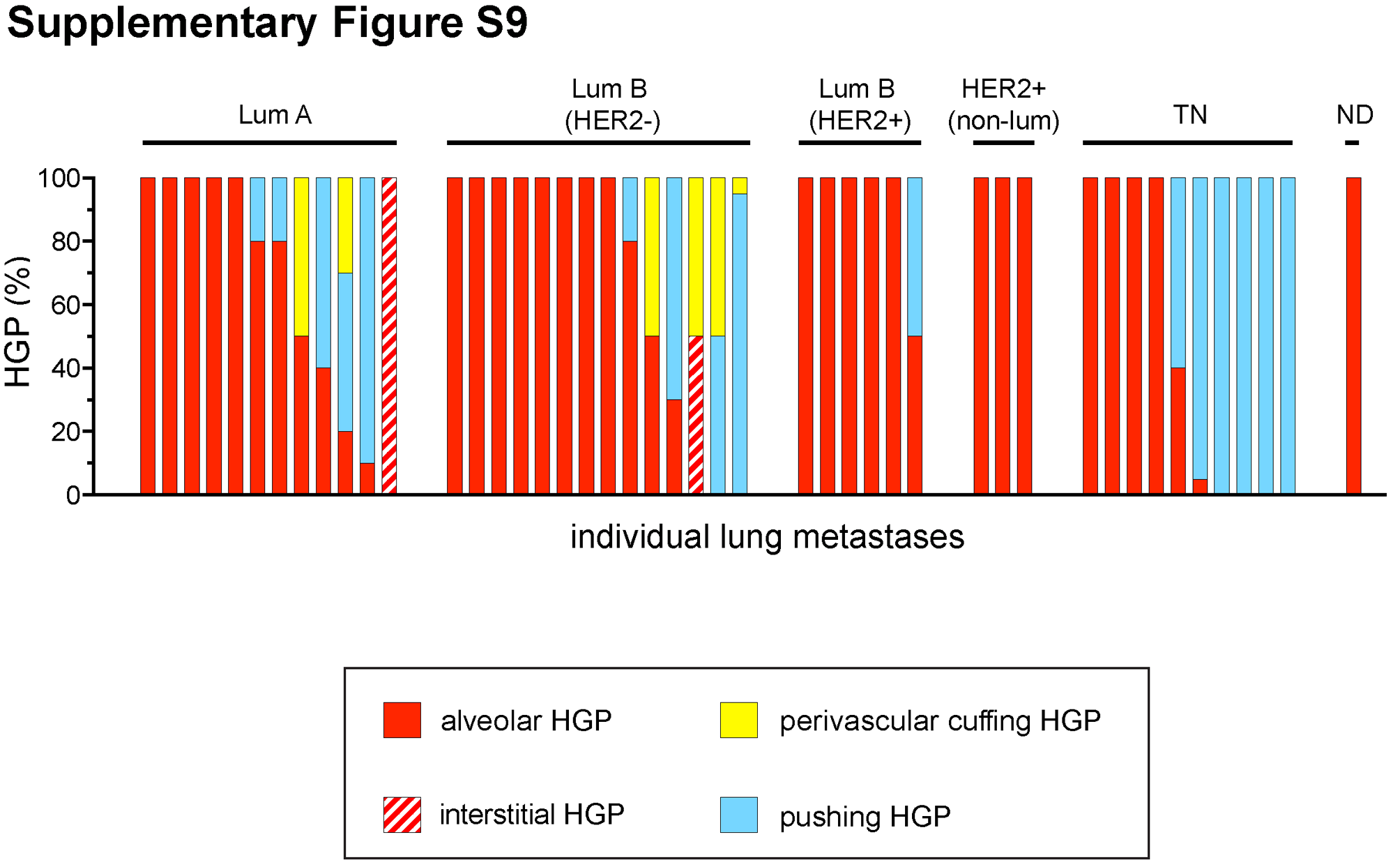

Supplement: Supplementary file 11 — Figure S9. Cases of breast cancer lung metastases grouped by intrinsic molecular subtype Graph shows the HGPs for 46 cases of breast cancer lung metastases (the same as scored in Figure 4A) grouped here by intrinsic molecular subtype. Lum A = luminal A; Lum B (HER2‐) = luminal B (HER2 negative); Lum B (HER2+) = luminal B (HER2 positive); HER2+ (non‐lum) = HER2 positive (non‐luminal) and TN = triple negative. For one of the cases we were unable to determine the subtype due to lack of sufficient tissue samples (ND, not determined). [file PATH-241-362-s011.tif]

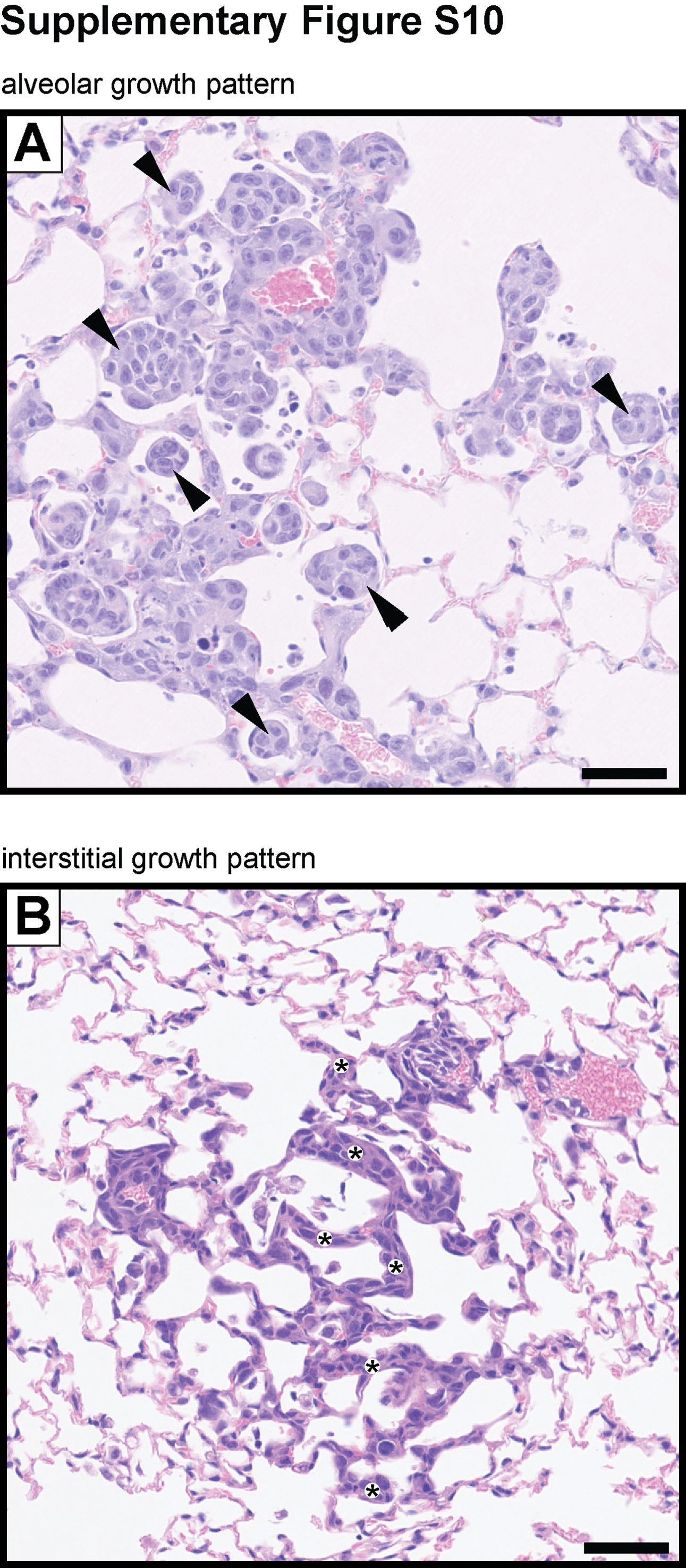

Supplement: Supplementary file 12 — Figure S10. High power views of alveolar growth pattern and interstitial growth pattern in preclinical lung metastasis models A. Alveolar growth pattern. In lung metastases formed by 4T1 cells, groups of 4T1 cells growing in alveolar air spaces are indicated with an arrowhead. B. Interstitial growth pattern. In lung metastases formed by C26 cells, groups of C26 cells that are growing within the alveolar walls are indicated with asterisks. Scale bar, 100 µm. [file PATH-241-362-s012.tif]

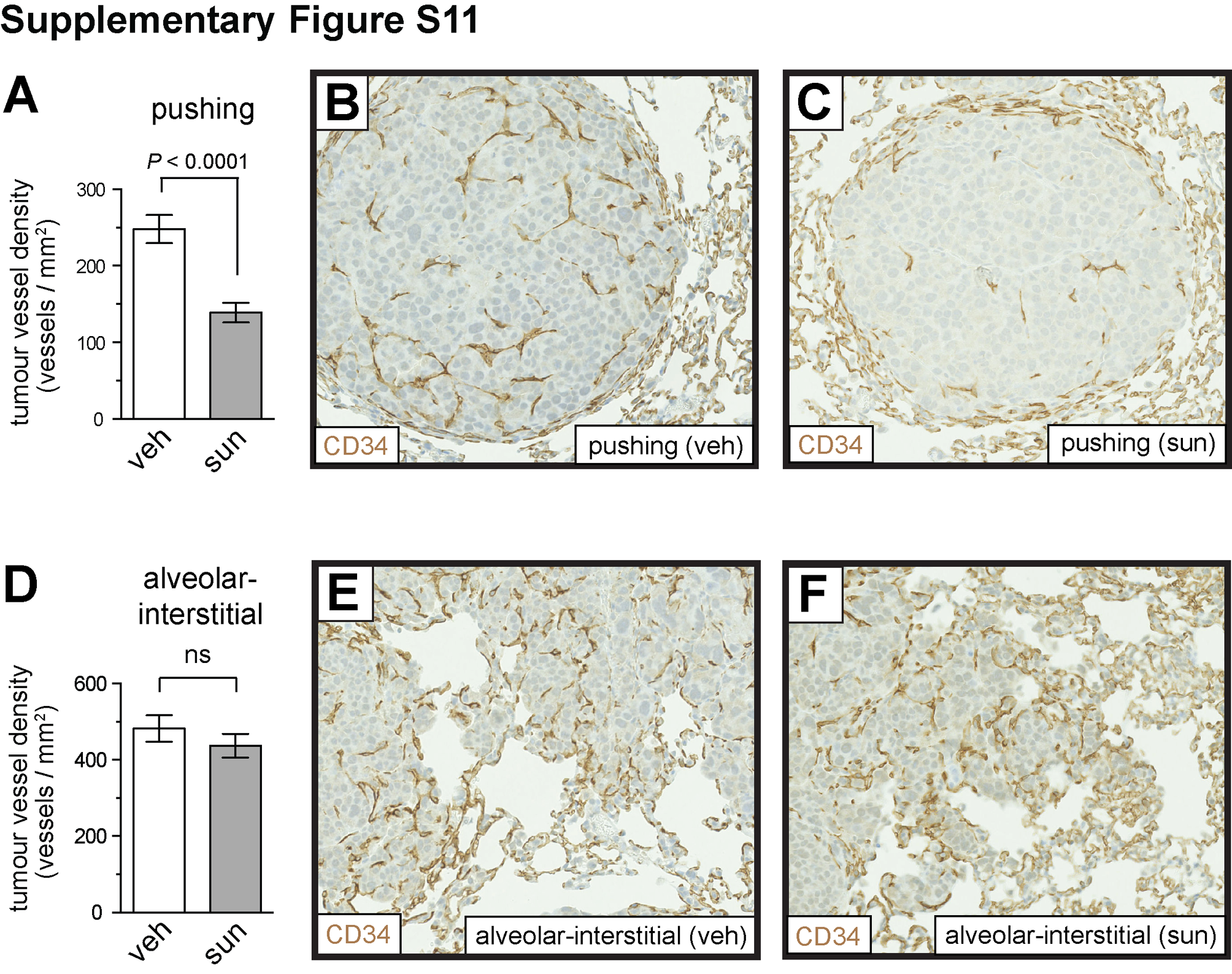

Supplement: Supplementary file 13 — Figure S11. Contrasting effect of sunitinib in RENCA lung metastases with different HGPs A‐F. Graphs show tumour vessel density +/− SEM in lung metastases from mice injected via the tail vein with RENCA cells and then treated for 10 days with either 40 mg/kg/day sunitinib (sun) or vehicle (veh) alone (A,D). Vessel density was quantified in pushing lung metastases (A) or alveolar‐interstitial lung metastases (D) separately. n = 20 lung metastases (from 5 mice) per data point. Representative images of CD34 staining in pushing lung metastases (B,C) and alveolar‐interstitial lung metastases (E,F) from vehicle (B,E) or sunitinib (C,F) treated mice are shown. No significant difference (ns). [file PATH-241-362-s013.tif]

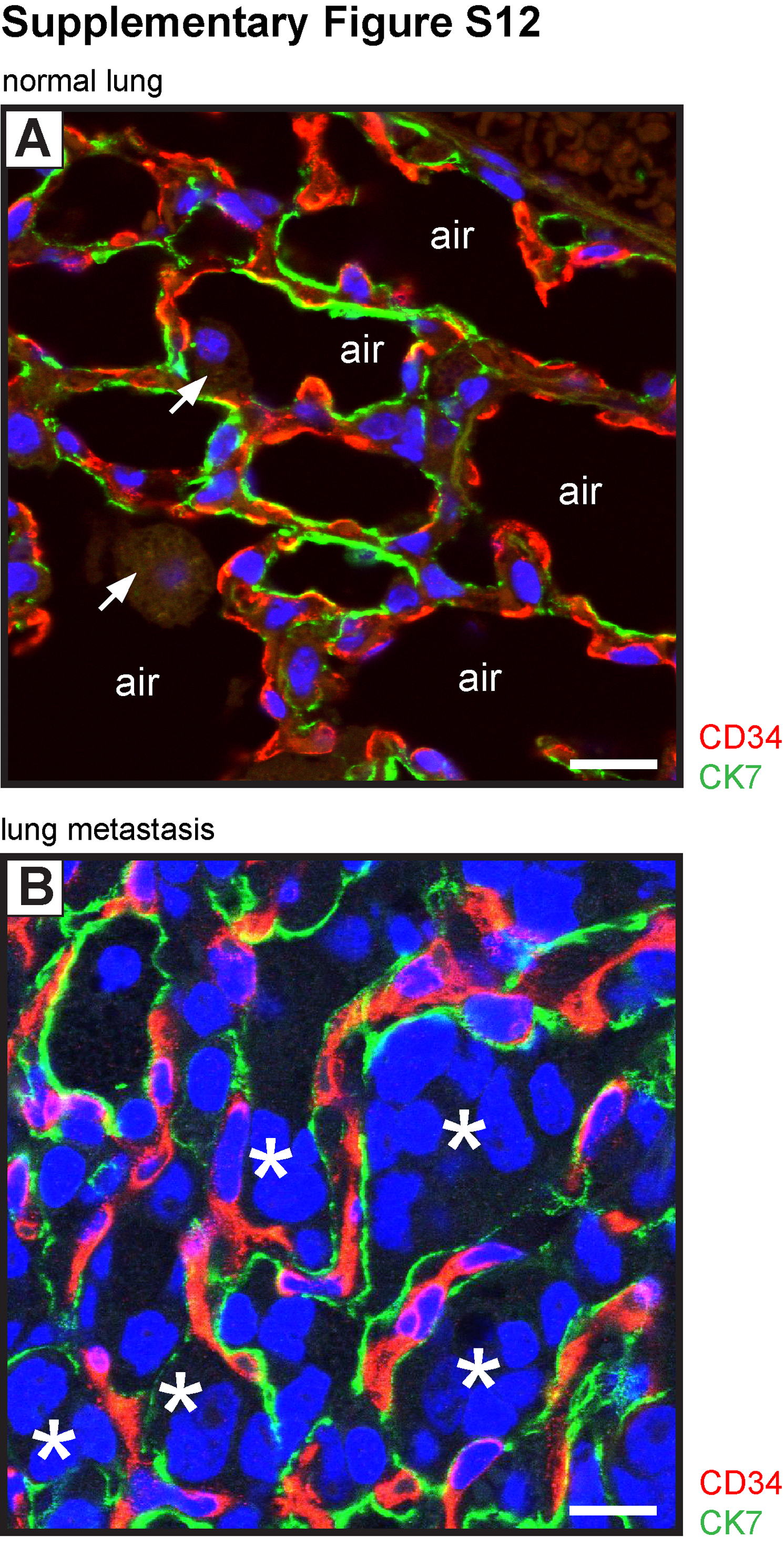

Supplement: Supplementary file 14 — Figure S12. Vessel co‐option in spontaneous MDA‐MB‐231LM2–4 lung metastases A,B. Staining for CD34, to demonstrate blood vessels (red), and cytokeratin 7 (CK7), to demonstrate pneumocytes (green), in normal mouse lung (A) or spontaneous lung metastases of MDA‐MB‐231LM2–4 cells (B). Note that the alveolar structure of the normal lung (A) is preserved within the lung metastases (B) indicating that the lung metastases in this model grow with an alveolar HGP and co‐opt pre‐existing alveolar capillaries. Asterisks indicate breast cancer cells present in the alveolar air spaces. Arrows indicate alveolar macrophages. Scale bar, 20 μM. [file PATH-241-362-s014.tif]
