# Supplementary material for: Vessel co‐option is common in human lung metastases and mediates resistance to anti‐angiogenic therapy in preclinical lung metastasis models
Source: J Pathol. 2016 Dec 29;241(3):362–74. doi: 10.1002/path.4845 (PMC5248628; doi:10.1002/path.4845)
Supplement: Supplementary file 15 — Table S1. Clinical characteristics of breast cancer patients Clinical characteristics of 46 patients with breast cancer lung metastases that were included in the study. Table S2. Clinical characteristics of colorectal cancer patients Clinical characteristics of 53 patients with colorectal cancer lung metastases that were included in the study. Table S3. Clinical characteristics of renal cancer patients Clinical characteristics of 59 patients with renal cancer lung metastases that were included in the study. [file PATH-241-362-s015.docx]

**Table S1. Clinical characteristics of breast cancer patients**

Clinical characteristics of 46 patients with breast cancer lung metastases that were included in the study.

| Gender, number of patients (%)  Male  Female | 2 (4.3)  44 (95.7) |
| --- | --- |
| Age when lung metastasis sample was obtained,  median (range) | 60 (30-82) |
| Source of sample, number of patients (%)  Surgical resection  Autopsy | 43 (93.5)  3 (6.5) |
| Size of lung metastasis analysed, number of lesions (%)  < 10 mm  10 – 19 mm  20 – 39 mm  ≥ 40 mm  N/A | 12 (26.1)  15 (32.6)  9 (19.6)  1 (2.1)  9 (19.6) |
| Systemic therapy received by patient prior to resection of lung metastasis, number of patients (%)*  None  Chemotherapy  Endocrine therapy  Bevacizumab  Herceptin  Bisphosphonate  N/A | 10 (21.7)  30 (65.2)  12 (26.1)  2 (4.3)  1 (2.2)  1 (2.2)  3 (6.5) |

**Footnote:**

N/A, accurate information was not available.

* Some patients received multiple lines of therapy.

**Table S2. Clinical characteristics of colorectal cancer patients**

Clinical characteristics of 53 patients with colorectal cancer lung metastases that were included in the study.

| Gender, number of patients (%)  Male  Female | 30 (57)  23 (43) |
| --- | --- |
| Age when lung metastasis sample was obtained,  median (range) | 63 (33-83) |
| Source of sample, number of lesions (%)  Surgical resection  Autopsy | 53 (100)  0 (0) |
| Size of lung metastasis analysed, number of lesions (%)  < 10 mm  10 – 19 mm  20 – 39 mm  ≥ 40 mm  N/A | 13 (22.8)  26 (45.6)  10 (17.5)  2 (3.5)  6 (10.5) |
| Systemic therapy received by patient prior to resection of the lung metastasis, number of patients (%)  None  Chemotherapy  Bevacizumab  N/A | 18 (34)  19 (35.8)  3 (5.7)  17 (32.1) |

**Footnote:**

N/A, accurate information was not available.

**Table S3. Clinical characteristics of renal cancer patients**

Clinical characteristics of 59 patients with renal cancer lung metastases that were included in the study.

| Gender, number of patients (%)  Male  Female | 42 (71.2)  17 (28.8) |
| --- | --- |
| Age when lung metastasis sample was obtained,  median (range) | 62 (30-77) |
| Source of sample, number of lesions (%)  Surgical resection  Autopsy | 58 (98.3)  1 (1.7) |
| Size of lung metastasis analysed, number of lesions (%)  < 10 mm  10 – 19 mm  20 – 39 mm  ≥ 40 mm | 14 (23)  19 (31.1)  18 (29.5)  10 (16.4) |
| Systemic therapy received by patient prior to resection of the lung metastasis, number of patients (%)*  None  Interferon  Chemotherapy  Sunitinib  Pazopanib  Sorafenib  Interleukin 2  N/A | 16 (27.1)  13 (22)  11 (18.6)  5 (8.5)  2 (3.4)  1 (1.7)  1 (1.7)  18 (30.5) |

**Footnote:**

N/A, accurate information was not available.

* Some patients received multiple lines of therapy.
